# Can any Basic/Nucleophile Quaternary Salt Promote the Carbonatation of Epoxides? A Review

**DOI:** 10.1021/acsomega.5c03990

**Published:** 2025-07-24

**Authors:** Brenno A. D. Neto, Roberto Y. de Souza, Jairton Dupont

**Affiliations:** † Laboratory of Medicinal and Technological Chemistry, Institute of Chemistry, 28127 University of Brasília, Campus Universitário Darcy Ribeiro, Distrito Federal, Brasília 70904-900, Brazil; ‡ Molecular Sciences Graduate Program, State University of Goiás, Anápolis, Goiás 75001-970, Brazil; § Brasília Military College (CMB—Colégio Militar de Brasília), 902/904, Asa Norte, Brasília, DF 70790-020, Brazil; ∥ Institute of Chemistry, 28124 Universidade Federal do Rio Grande do Sul, Av. Bento Gonçalves, 9500, Porto Alegre, RS 91501-970, Brazil

## Abstract

This review examines the role of ionic liquids (ILs) in the catalytic carbonation of epoxides for the synthesis of cyclic carbonates, focusing on the key factors that influence reaction efficiency. The nucleophilicity and basicity of the anions in IL catalysts are highlighted as critical components for promoting the cycloaddition reaction with CO_2_. The solubility and ionicity of the ILs also significantly affect the reaction, with higher ionicity leading to better solubilization and catalytic performance. Additionally, the review emphasizes the importance of the water content in influencing solvation and the generation of nucleophilic species, particularly in hydrophilic IL environments. Mechanistic studies, supported by density functional theory (DFT) calculations, provide insight into the reaction pathways, although more detailed kinetic data are required. The induction period observed in some reactions suggests the formation of ionic nucleophilic species, but it is still premature to draw definitive conclusions regarding the effects of various reaction parameters. The review calls for further exploration of the kinetic data and competitive experiments to fully understand the role of ILs in epoxide carbonation.

## Graphical abstract

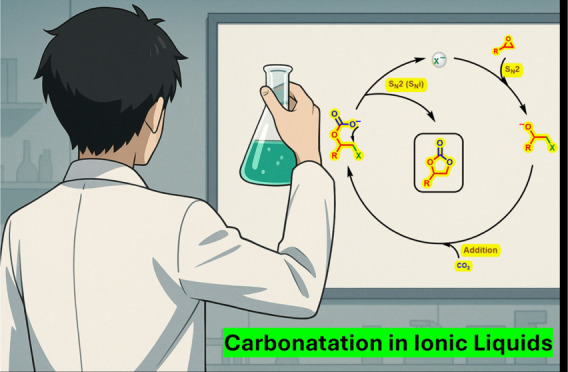

## Initial Considerations

The carbonation reaction of epoxides, leading to the formation of cyclic carbonates, is a focal point in extensive research within the carbon dioxide valorization domain. These cyclic carbonates find diverse industrial applications, serving as versatile polar solvents and essential precursors for polycarbonate materials.^1–3^ Additionally, they play vital roles in lithium batteries as electrolytes,^4^ contributing significantly to advancements in energy storage technologies. Integral to pharmaceutical production, cyclic carbonates are key participants in the synthesis of pharmaceutical compounds.^5^ Their versatile chemistry and compatibility make them valuable raw materials in various chemical processes, contributing to the synthesis of compounds and materials with diverse applications. The carbonation of epoxides and the subsequent formation of cyclic carbonates have become crucial elements in the exploration of sustainable and industrially relevant processes involving carbon dioxide.^6–8^ Various technologies exist for utilizing CO_2_ as a raw material across different Technology Readiness Levels (TRL 1 to 9), ranging from CO_2_ reduction to produce methanol to the synthesis of CO_2_-based polycarbonates, the latter of which are already commercially available.^9^

There are literally hundreds of contributions in this field, especially regarding the use of quaternary (mainly ammonium and phosphonium) salts as catalysts for incorporating carbon dioxide. Additionally, there are excellent reviews published in recent years.^10–44^ In particular, the outstanding contributions of M. North and co-workers have emphasized various mechanistic, electronic, and steric effects, along with applications of both homogeneous and heterogeneous catalysts/catalysis involved in the carbonation of epoxides.^45^ In fact, nearly any quaternary (mostly nitrogen or phosphorus)-based compound containing a basic/nucleophilic anion can readily facilitate the carbonation of monosubstituted epoxides under relatively mild reaction conditions (temperature and CO_2_ pressure). One of the first examples involves the carbonation of ethylene oxide, promoted by phosphonium iodide salt, to generate the ethylene carbonate as an intermediate that is used to produce ethylene glycol (Scheme 1), patented by Mitsubishi several years ago.

**Scheme 1:**
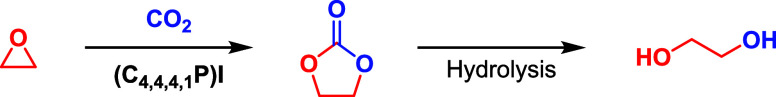
Carbonation of Ethylene Oxide Catalyzed by [C_4,4,4,1_P]­I Followed by Hydrolysis to Generate Ethylene Glycol

However, despite the abundance of available data, several aspects of the carbonation of epoxides remain unclear. In fact, many contributions overlook important effects that can influence the outcome of this reaction. Comparing the activity and selectivity of different precatalysts is nearly impossible due to their operation under vastly different reaction conditions (homogeneous/heterogeneous conditions, CO_2_ pressure, temperature, and time, as seen in Tables 1–4). Some of these aspects that should be considered before a proper comparison of catalytic performance are listed below:

1. Homogeneous and heterogeneous catalytic reaction conditions: single phase or multiphase.
2. Solubility of the precatalyst in the reaction medium comprising epoxide and CO_2_ conditions.
3. Homogeneous or heterogeneous catalysts: single site or multisite.
4. Nucleophilicity/basicity: the influence of solvation conditions and water content.
5. Yields and conversions are not appropriate to measure catalytic activity.

**Table 1:** Comparison of p*K*_a_ and Nucleophilicity (*N*) in Water of Selected Anions

entry	anion	p*K*_a_	*n*
1	I^–^	–10	5.0
2	Br^–^	–9	3.5
3	Cl^–^	–7	2.7
4	PhO^–^	10	3.5
5	HO^–^	15.7	4.2

We will critically discuss those aspects by analyzing the data available and present the scope and limitations of the carbonation of epoxides.

## Carbonation Reactions in ILs

Yield and substrate conversion are not always sufficient measures of catalytic activity as they do not always provide a comprehensive overview of the catalyst’s performance. While these metrics can be valuable in certain scenarios, they may not fully represent the efficiency, selectivity, or overall effectiveness of a catalyst in specific reactions. Several reasons why yield and substrate conversion may fall short include considerations of selectivity, catalyst loading, product purity, reaction conditions, kinetics, and mechanistic insights. In reality, catalytic reactions often entail multiple steps, each with varying kinetics. Yield and conversion metrics may overlook the complexities of these kinetic processes. For instance, a catalyst might efficiently convert a substrate to an intermediate but struggle with subsequent steps that lead to the desired product. In addition, comprehending the mechanistic intricacies of a catalytic reaction is vital for optimizing the catalyst design. Yield and substrate conversion metrics offer limited insight into the reaction mechanism, making it challenging to make informed decisions for catalyst improvement.

While various reaction pathway variants exist, all carbonation reactions of epoxides primarily involve two fundamental mechanisms through nucleophilic substitution reactions: S_N_2 and S_N_i (Scheme 2). The S_N_2-type mechanism is the predominant pathway for most epoxides, with the notable exception of methylene-hydroxide-substituted ones, such as glycidyl epoxides, which preferentially undergo the S_N_i mechanism. In both cases, the reaction is contingent on the nucleophilicity and basicity of the catalyst. It is noteworthy that both “nucleophilic” and “basic” imply a tendency to supply an unshared pair of electrons to form a new covalent bond. However, “nucleophilic” is employed in discussions of rate, whereas “basic” pertains to equilibrium conditions. For instance, Br^–^ and PhO^–^ exhibit the same nucleophilicity (≈3.5), yet the p*K*_a_ values illustrate a significant difference: HBr has a p*K*_a_ of −9, while phenol is much more basic, with a p*K*_a_ of 10. Therefore, it is crucial to emphasize that the comparison of the catalytic performance (kinetic) of a given catalyst should not be conducted under equilibrium conditions.^46^ Table 1 presents a selection of anions along with their corresponding p*K*_a_ values and nucleophilicities.

**Scheme 2:**
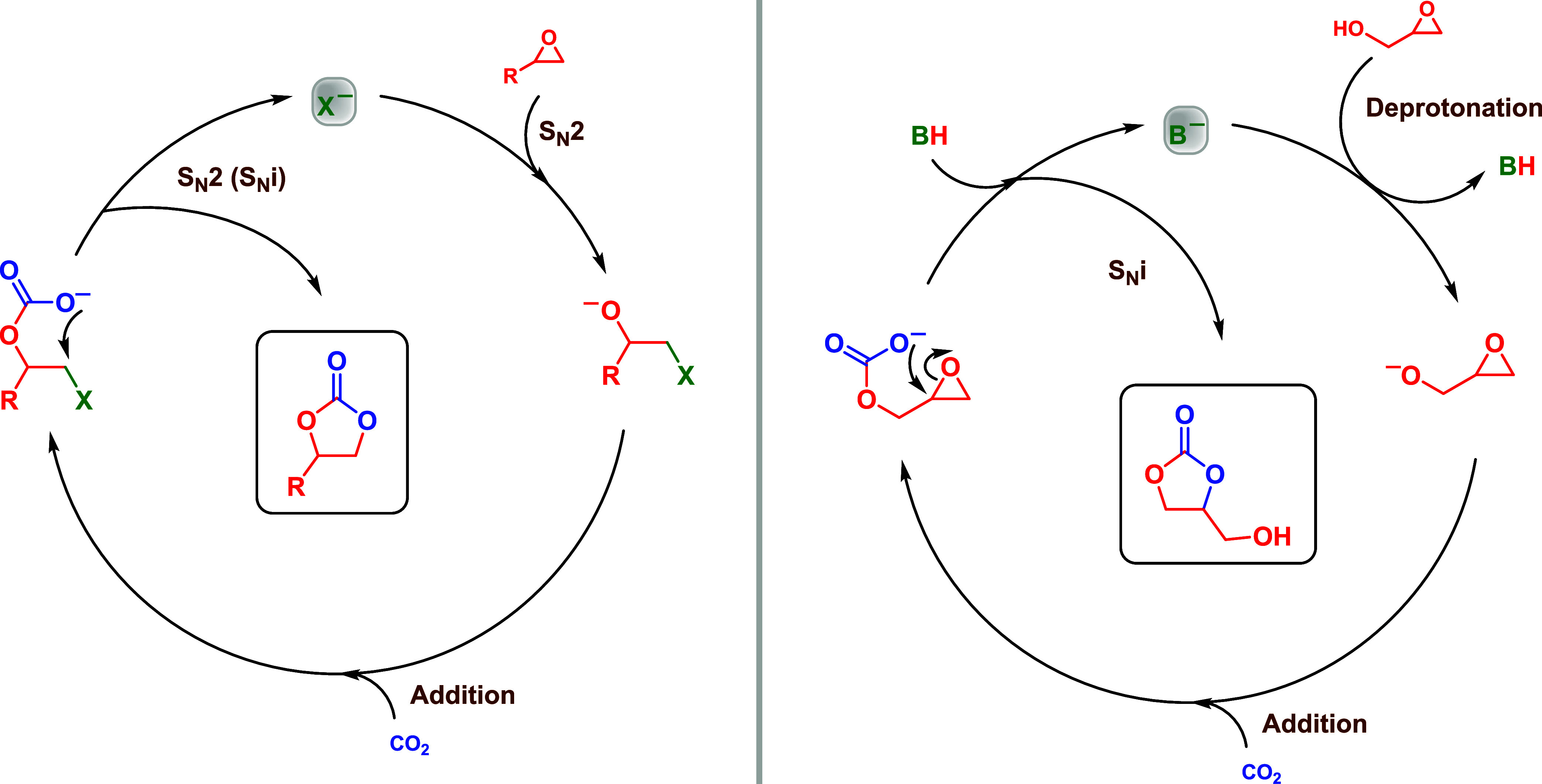
Catalytic Reaction Pathways Involved in the Carbonation of Epoxides^a^

a (Left) S_N_2 reaction in which X^–^ behaves as a nucleophile. (Right) S_N_i reaction in which B^–^ acts as a base.

**Scheme 3:**
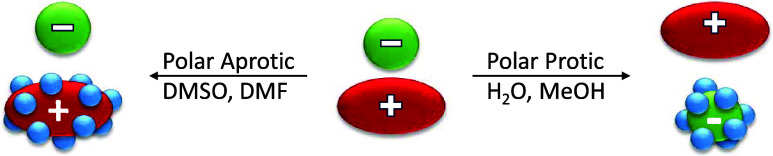
Solvation Models Considering Polar and Protic, as well as Polar and Aprotic Solvents, for a Generic Ion Pair

It is beneficial to recall some fundamental principles: (i) Among species of a given element, negatively charged entities exhibit greater nucleophilicity (and basicity) compared to their neutral counterparts. (ii) Across a given period of the periodic table, nucleophilicity (and basicity) tends to decrease when progressing from left to right. (iii) Within a specific group of the periodic table, nucleophilicity generally increases from top to bottom, corresponding to an increase in size, although solvent dependence may arise due to hydrogen bonding. Basicity, however, follows the opposite trend. The nucleophilicity of a nucleophile is also influenced by the reaction medium. In methanol, for instance, the nucleophilicity of some common Nu:(−) reactants varies as indicated: CH_3_CO_2_(−) < Cl(−) < Br(−) < N_3_(−) < CH_3_O­(−) < CN(−) ≈ SCN(−) < I(−) < CH_3_S­(−).

Polar and protic solvents such as water and alcohols solvate anions through hydrogen bonding interactions, rendering these solvated species more stable and less reactive compared to their unsolvated “naked” counterparts. On the other hand, polar and aprotic solvents like DMSO (dimethyl sulfoxide), DMF (dimethylformamide), and acetonitrile, while not as proficient in solvating anions as methanol, exhibit effective solvation of accompanying cations (Scheme 3). Consequently, solutions prepared from these solvents facilitate faster reactions for the nucleophiles discussed here. These solvent effects are particularly pronounced for small, basic anions compared to larger, weakly basic anions. In DMSO solutions, the reactivity order is as follows: Nucleophilicity—I(−) < SCN(−) < Br(−) < Cl(−) ≈ N_3_(−) < CH_3_CO_2_(−) < CN(−) ≈ CH_3_S­(−) < CH_3_O­(−). Notably, this order roughly aligns with increasing basicity.

The quantitative correlation of relative rates nucleophilic reagents toward epoxides (epichlorohydrin and glycidol, for example) in water was established over a half a century ago and clearly shows the order of nucleophilicity Cl(−) < Br(−) < I(−) (Figure 1).^47^

**Figure 1:**
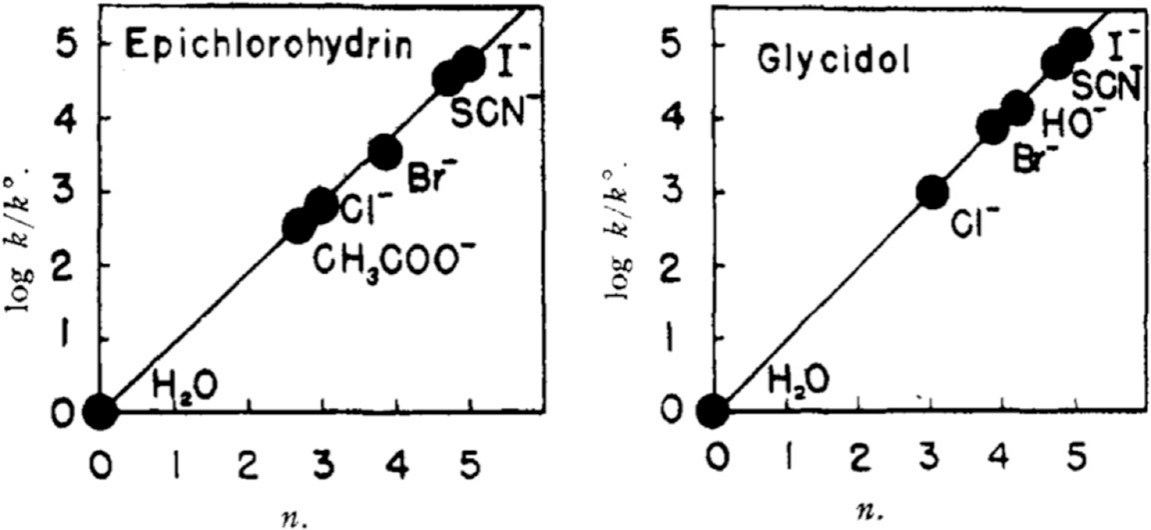
Plots of log *k/k* ^o^ vs nucleophilicity reactivity for anion addition to epichlorohydrin and glycidol in water. Adapted from reference.^47^ Copyright 1953 American Chemical Society.

Solvation and solubilization are distinct processes that occur when organic salts dissolve in a solvent. Solvation involves solvent molecules surrounding and interacting with individual ions of the solute, breaking the ionic lattice of the salt, and dispersing its constituent ions throughout the solvent. This process is typically endothermic because energy is needed to overcome the electrostatic forces holding the ions in the crystal lattice and to separate the solvent molecules for ion–solvent interactions.

On the other hand, solubilization occurs when molecules or ions that are typically insoluble or sparingly soluble in a solvent become dispersed or dissolved in solution due to the presence of surfactants, micelles, or other solubilizing agents. Unlike solvation, where the solute consists of ions held together by ionic bonds, solubilization may involve molecular compounds or organic salts lacking strong ionic bonds.

Solvation is primarily driven by the desire to stabilize the separated ions through ion-dipole interactions with the solvent molecules. In contrast, solubilization is driven by various factors, including entropy changes, solute–solvent interactions, and the formation of micelles or other aggregates. While solvation involves the dissociation of the salt into individual ions, solubilization entails the dispersion or dissolution of solute molecules or ions through the action of solubilizing agents. While solvation is generally endothermic due to the breaking of ionic bonds, solubilization can be either endothermic or exothermic depending on the specific interactions involved in the process. In summary, while both solvation and solubilization entail the dispersion of solute species in a solvent, they differ in terms of the nature of the solute, driving forces, mechanism, and energy changes associated with the process.

While quaternary organic salts may solubilize at the onset of the reaction when in contact with the epoxides/CO_2_ mixture, it is improbable that they effectively undergo ion solvation, except in the cases of functionalized epoxides such as glycidine, epichlorohydrin, or styrene oxide. Quaternary organic salts, especially those based on the imidazolium cation, are structured as polymeric supramolecules formed via an extended and cooperative hydrogen bond network. The dissolution of ILs involves the formation of floating neutral and ionic aggregates (eq 1) of the type

1 $${\left\{{\left[\mathrm{cation}\right]}_{n}^{x}{\left[\mathrm{anion}\right]}_{n-1}^{-x}\right\}}^{+}\mathrm{or}{\left\{{\left[\mathrm{cation}\right]}_{n}^{x}{\left[\mathrm{anion}\right]}_{n+1}^{-x}\right\}}^{-}$$

Even in solvents with polar (protic and aprotic) dielectric constants, and the formation of solvent-separated ions is limited (see Scheme 4).^48^ The solubilization and solvation of organic salts are facilitated in the case of epoxides containing polar groups such as epichlorohydrin and glycine, while they are less pronounced in the case of nonfunctionalized epoxides. Indeed, it was recently demonstrated that high yields depend heavily on catalyst solubility in the reaction medium, but solubility alone does not guarantee reaction success and was suggested that catalyst dissociation, reliant on solubility, is a critical factor in defining the catalytic activity.^49^

**Scheme 4:**
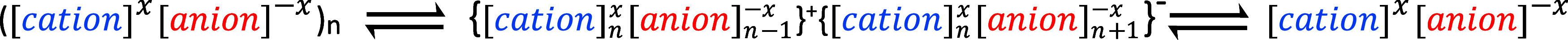
Supramolecular Aggregates Formation Observed in ILs during Their Dissolution

It is quite plausible that at the beginning of reactions performed under solventless conditions, the nonfunctionalized epoxides/CO_2_ mixture will result in very low (if any) concentrations of anionic nucleophilic catalytically active species. In the case of “pure” salts, it is much more probable for the presence of anionic nucleophilic aggregates than isolated ions, a condition that depends on the ionicity of the salt.^50^

Ionicity in ILs is directly related to many physicochemical properties, such as viscosity, conductivity, and osmotic pressure, making it an essential characteristic of these ionic fluids, although it is sometimes overlooked. The concept and measurement of ionicity for ILs have been discussed elsewhere^51–53^ From a microscopic perspective, ion pairing in pure ILs can be understood by considering both distance and time scales. This involves determining how far and for how long oppositely charged ions remain close and move together within the liquid.^54^ Molecular dynamics simulations have demonstrated that ion pairs in ILs are very short-lived and the ions tend to diffuse mostly independently of each other, suggesting minimal ion pairing. Consistent with this observation, later studies have shown that separating ion pairs requires relatively little energy, which aligns with the charge screening effect observed in ionic environments.^55^

In this context, ionicity plays an essential role, as it is a property directly associated with anion availability, which is, in turn, closely related to the nucleophile concentration in halogenated ILs. Given that nonfunctionalized (nonpolar substances) epoxides and CO_2_ may not effectively solvate the organic salts to generate anionic nucleophilic catalytically active species, it is plausible that these species may be generated by the residual water invariably present in these salts. Indeed, organic salts are generally highly hygroscopic, and the presence of water will generate nucleophilic catalytic species. The ionicity of charged species in water has been studied far more extensively than in the complex nanostructures of “pure” ILs. Therefore, the presence of residual water may increase the availability of halogens (anions) to promote the carbonation reaction.

It is essential to acknowledge that most organic salts typically contain significant amounts of residual water.^56^ For instance, imidazolium salts generally contain at least 7 water molecules per ionic pair.^57^ This residual water content can influence the reaction dynamics and should be considered in evaluating the nucleophilic behavior of the anions in epoxides. Additionally, the interaction of CO_2_ with organic salts, especially 1,3-dialkyimidazolium-based ILs, primarily occurs at the nonpolar domains of the ionic liquid (IL). This interaction neither disrupts the cation–anion interaction nor competes with the water interaction sites present at the polar domains.^58^

It is important that for “pure” ILs, primarily those based on the imidazolium cation, the ion dissociation decreases with the increasing alkyl chain length, thereby increasing the van der Waals interactions. Additionally, ILs whose charges are more delocalized on the anion will exhibit greater ion dissociation.^50^ Therefore, the formation of ionic nucleophilic species in pure organic salts should follow the order of ionicity, i.e., I(−) > Br(−) > C­(l)–.

However, despite the plethora of reports, the purity and water content of organic salts are rarely reported, making it challenging to draw clear conclusions. It is worth noting that although the data is based on yields/conversions (see Table 1), the reported data indicate that the yield/conversion follows the same order of nucleophilicity of the halides in water (Cl < Br < I). It was reported that the catalytic performance of supported ionic liquid-silica precatalysts increases with the addition of water, and this was attributed to halide solvation by water.^59^

Experimentally, the presence of anionic aggregates has been reported (Figure 2), such as ZnCl_3_^–^ and [BMIm]­[Fe­(NO)_2_Cl_2_] in the case of chloro-organo-zincate^60^ and ferrates^61^ indicating once more the dependence on ionic liquid’s ionicity.

**Figure 2:**
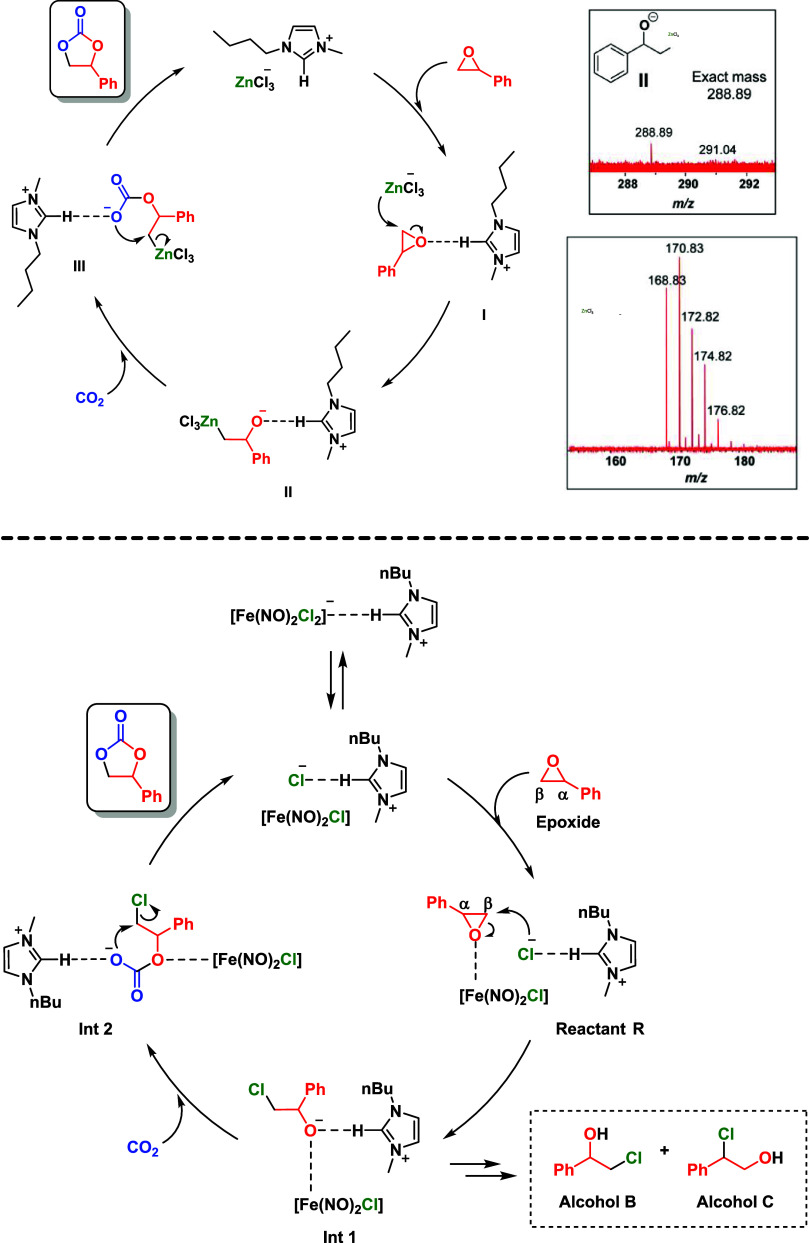
(Top) Anionic species observed in the carbonation of epoxides by organo-zincate and the proposed catalytic cycle and (Bottom) iron-catalyzed reaction.

There are numerous solid catalysts utilized in the carbonation of epoxides, including polymeric ILs, supported ionic liquid phase catalysts (SILP), and grafted quaternary salts on organic and inorganic solid supports. In all of these cases, the material likely functions as a “catalyst delivery device”, as the reaction is promoted by the nucleophiles (generally halides) released into the solution. The catalytic active sites are well-known, and, in most instances, these sites consist of single “atoms” that are likely solvated. The reaction is assumed to be typically homogeneous, occurring in solution rather than at the support surface, except in cases where the surface may act as a Lewis acid, activating the epoxide.

It is noteworthy that the real catalytic active species may vary even for “homogeneous” conditions since it will depend on the ion dissociation under the experimental conditions employed. Therefore, the TON and TOFs reported so far may be underestimated as large anionic aggregates may be catalytically active along with “isolated” anions. Without real kinetic studies, it is premature to assign and compare the catalytic activity of the hundreds of salts investigated so far.

As already stressed, nucleophilicity depends on the reaction medium, especially the solvent,^62^ which can be either polar and aprotic or polar and protic. However, considering that most reactions are conducted under solventless conditions and CO_2_ pressure, there is a limited understanding of anion nucleophilicity in epoxides. It can be hypothesized that, in such conditions, anions may exhibit weaker interactions with the cation, potentially enhancing the anion’s nucleophilicity.

The reported data on the carbonation of epoxides primarily focus on epoxide conversions and cyclic carbonate yields. It is asserted that the combination of the organic halide and a Lewis acid compound can significantly influence the overall performance of the catalytic system in the cycloaddition of carbon dioxide to epoxides.^14,63,64^ The type of halide used as a nucleophile and the cation in the organic halide salt both play crucial roles in defining the catalytic activity and selectivity of the system.^14^ However, in most of these studies, the analysis of the reactivity is conducted at higher epoxide conversions. Additionally, the reaction is typically performed under solventless conditions where the epoxide serves as the “solvent”. Therefore, under these conditions, the cation will be preferentially solvated.

Additionally, only a few cases have taken into consideration the presence of water, which solvates the anion in the catalyst precursor. Note that it was reported in various cases that the addition of water or alcohols (e.g., Table 2, entries 1, 60, and 62) increases the catalytic performance of the organic salt catalyst precursors. In these cases, the reaction kinetic profile should present an induction period, i.e., reflecting the ion solvation.^65–84,145–416^

**Table 2:**
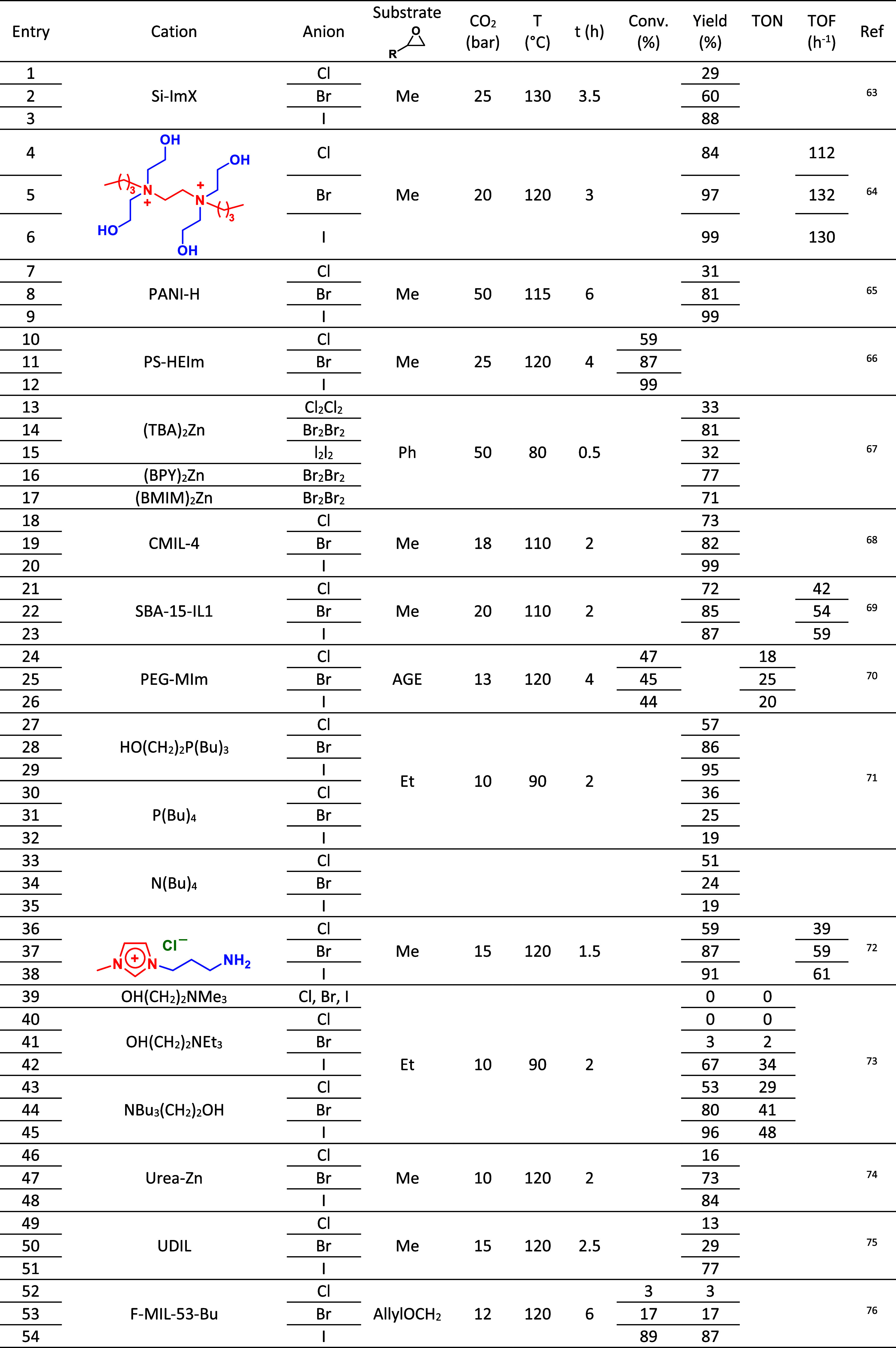

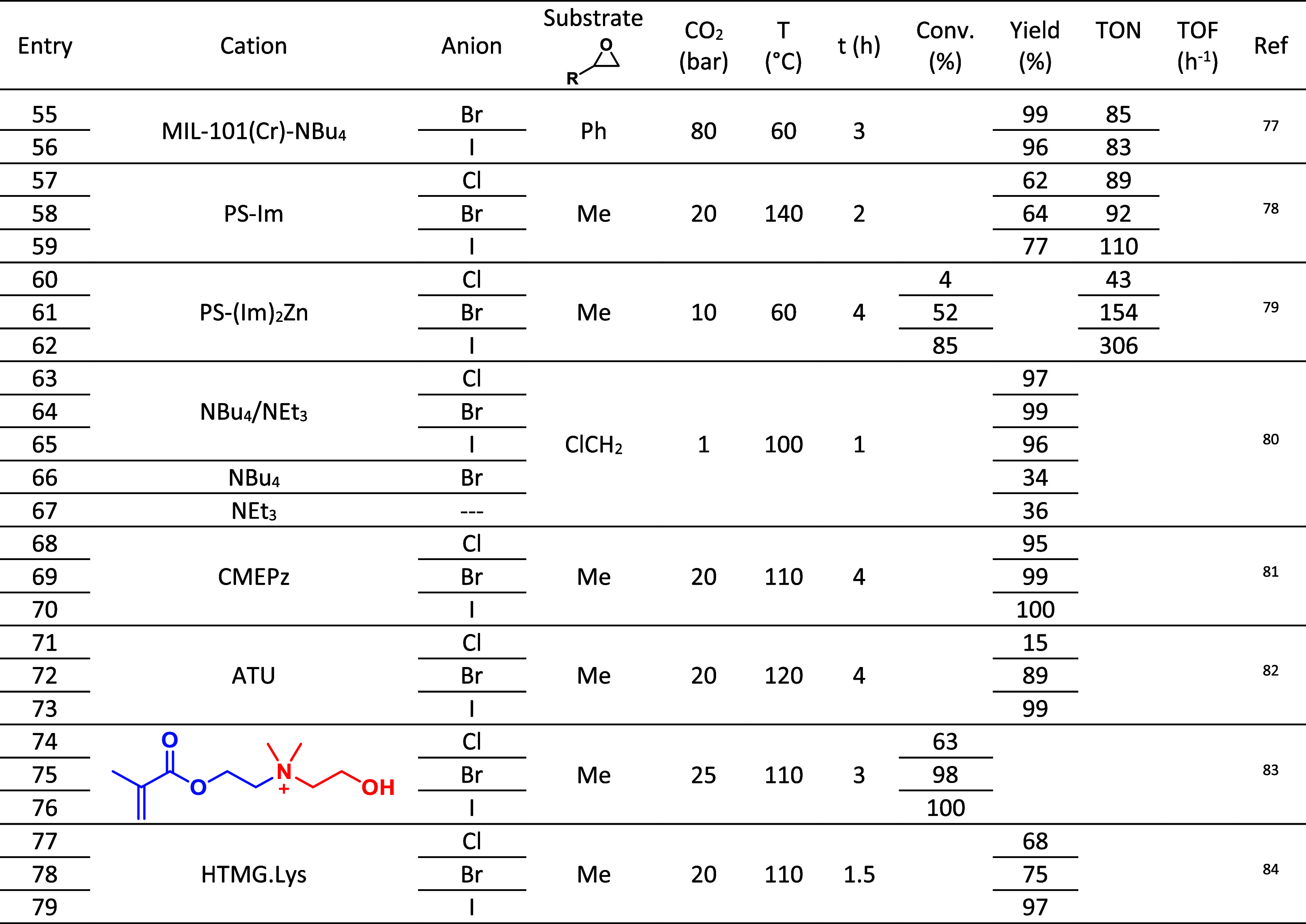
Anions Based on Halogens and the Reaction Conditions for Carbonation from Epoxides

Nucleophilicity of the halides and ionicity follows the order Cl < Br < I, consistent with the expected trend for these nucleophiles in polar protic solvents. However, since the reactions are conducted under solventless conditions (i.e., the epoxides/CO_2_ medium), it is evident at first glance that they are solvating the anions. This is contrary to what one might anticipate, given that both epoxide/CO_2_ are located in the nonpolar zones of the salts. This suggests that the employed salts likely contain significant amounts of water, responsible for anion solvation, a feature not usually recognized. Indeed, it is well-known that most organic quaternary salts contain residual water, even in highly hydrophobic ones. The leaving ability follows the order I > Br > Cl for these halogens. Consequently, it can be deduced that the leaving ability rather than the nucleophilic ability is dominant for the activity (Table 3). However, it should be stressed that due to the limited experimental data available this conclusion should be taken in caution. Only with more detailed kinetic studies more accurate conclusions can be made.

**Table 3:**
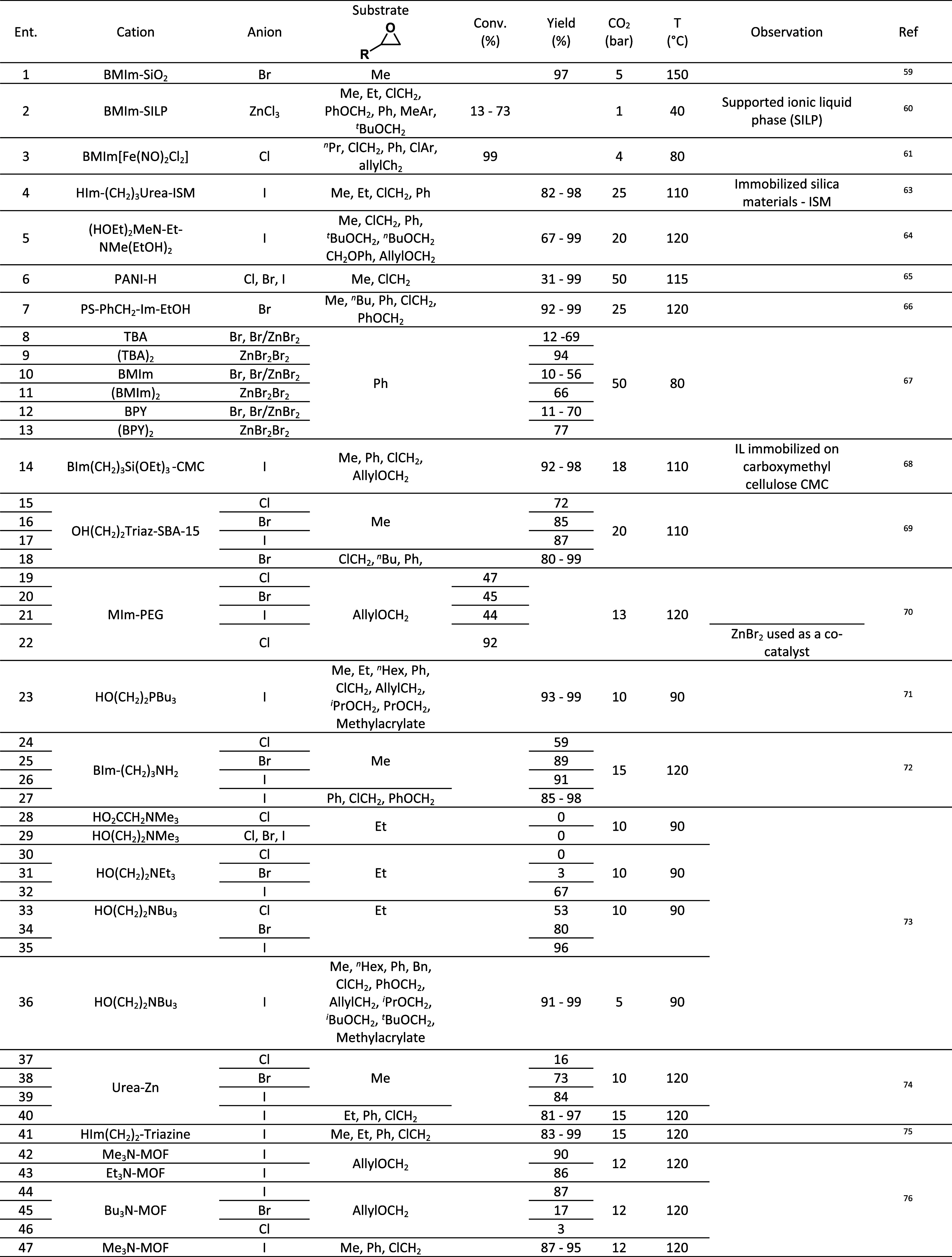

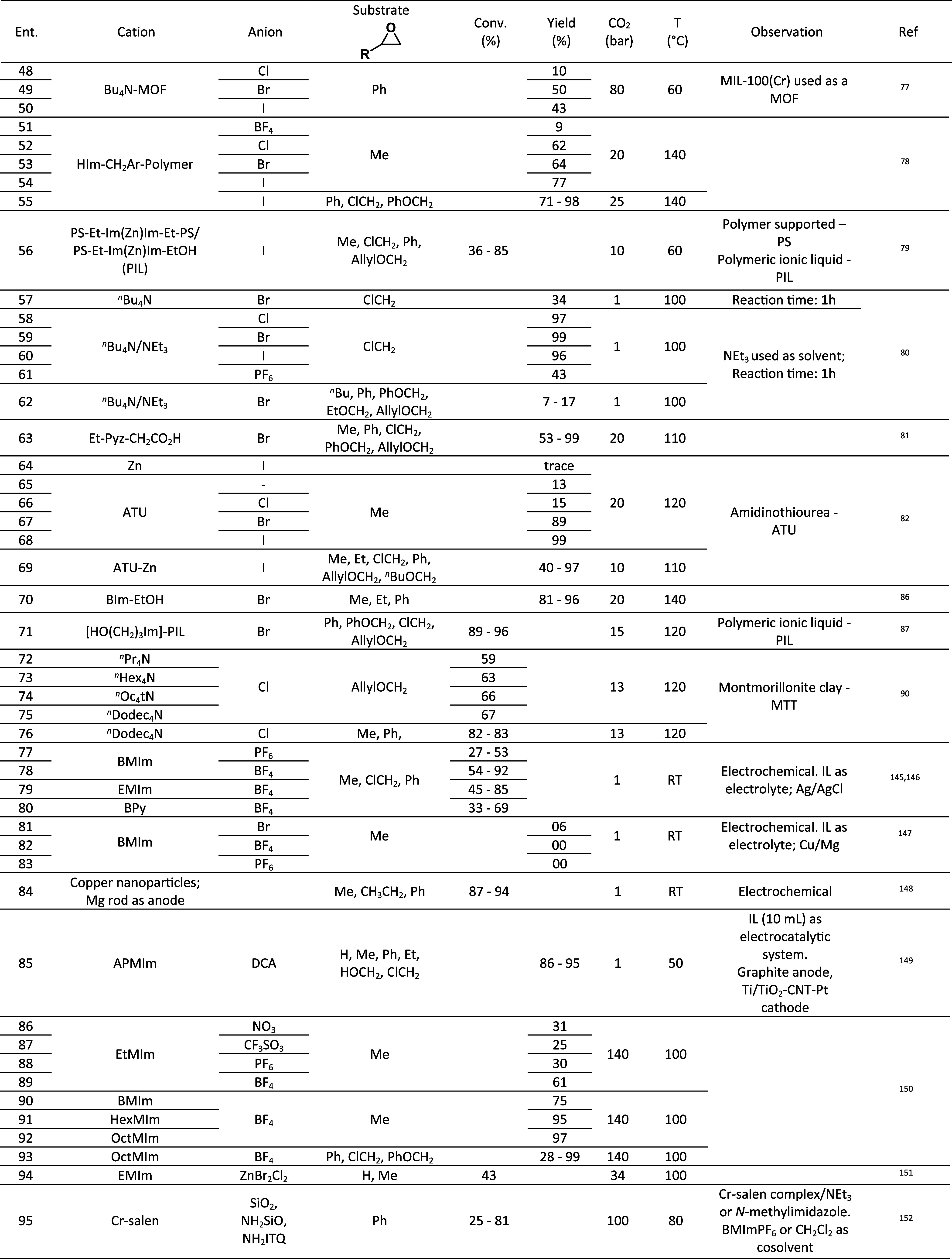

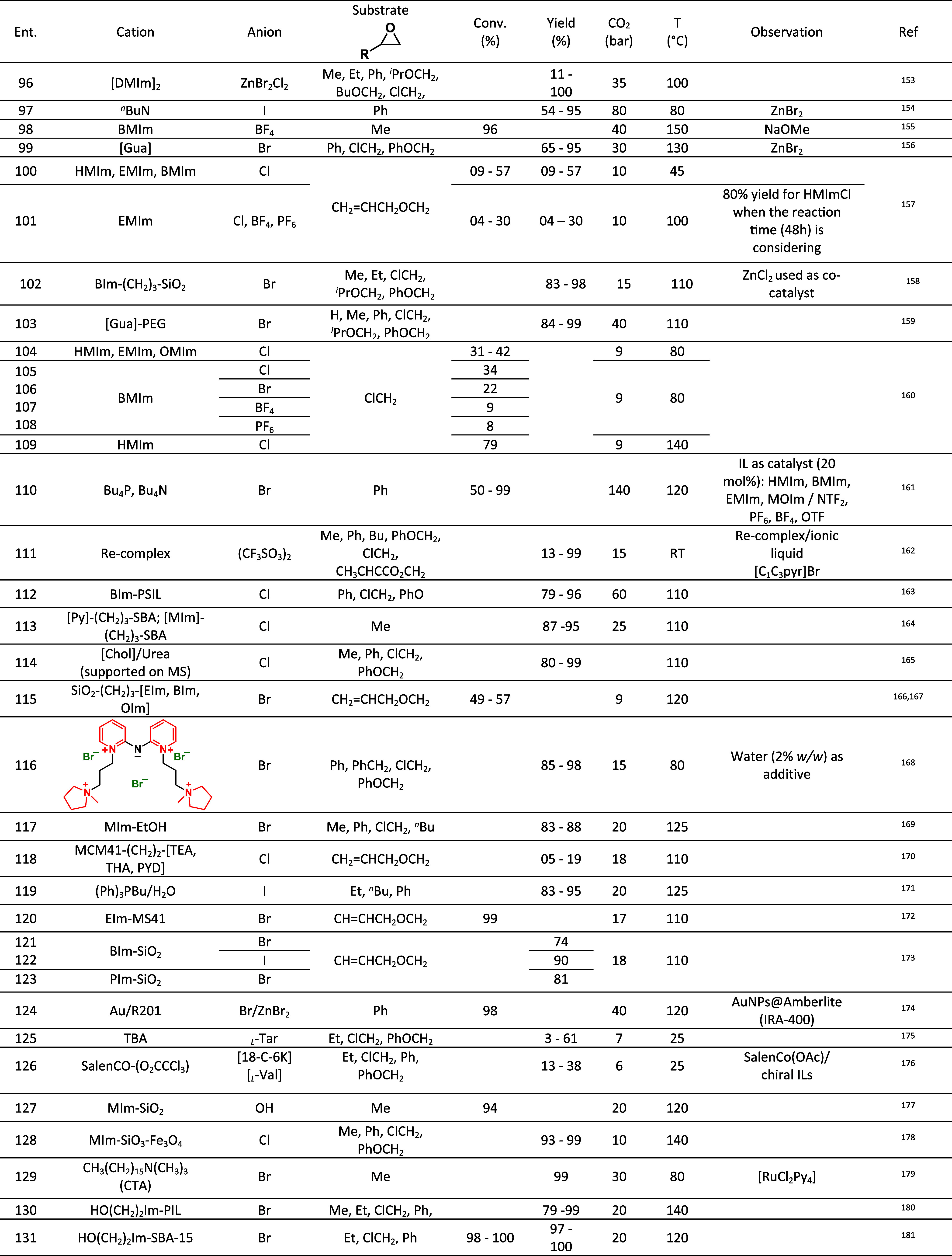

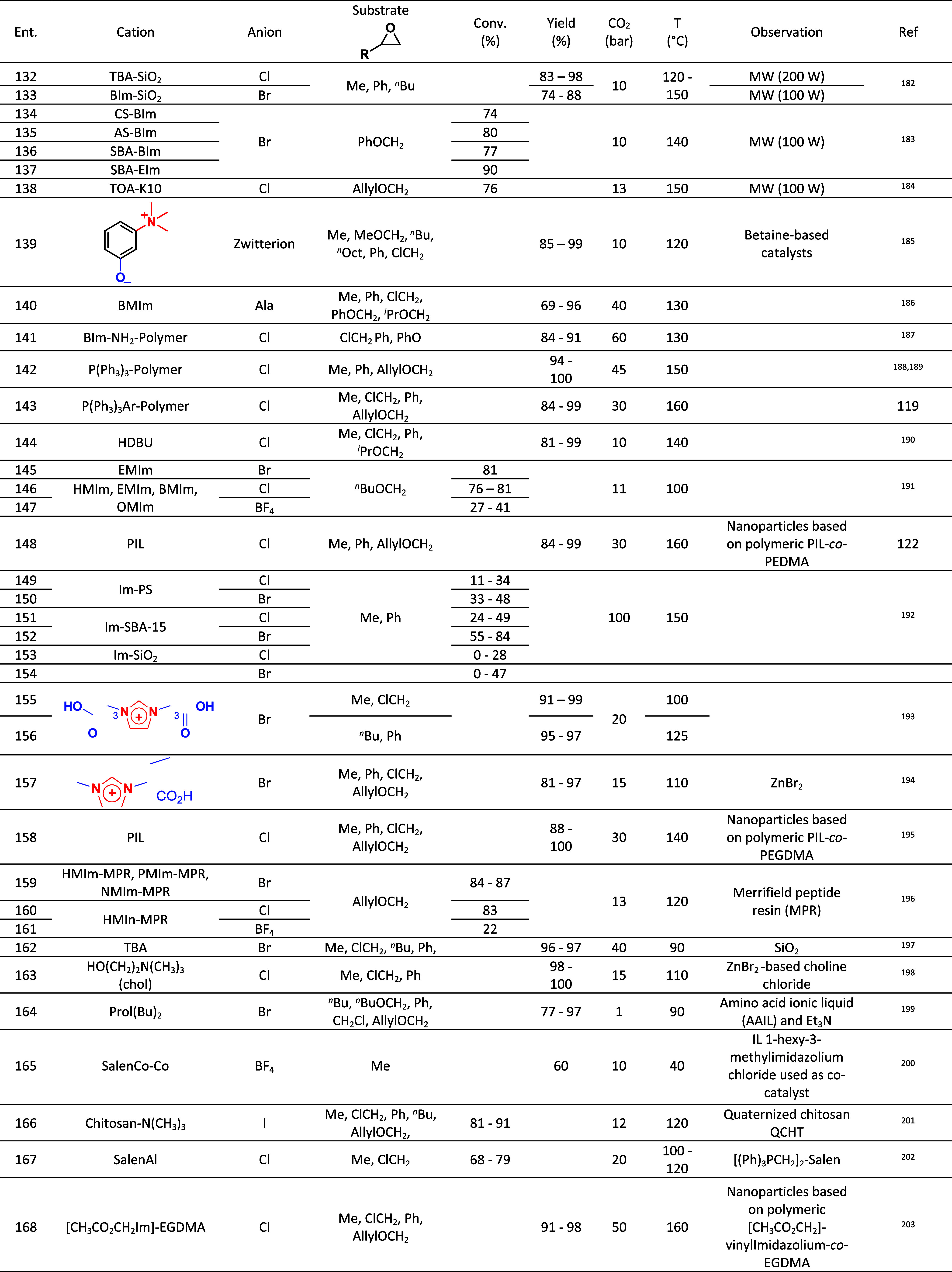

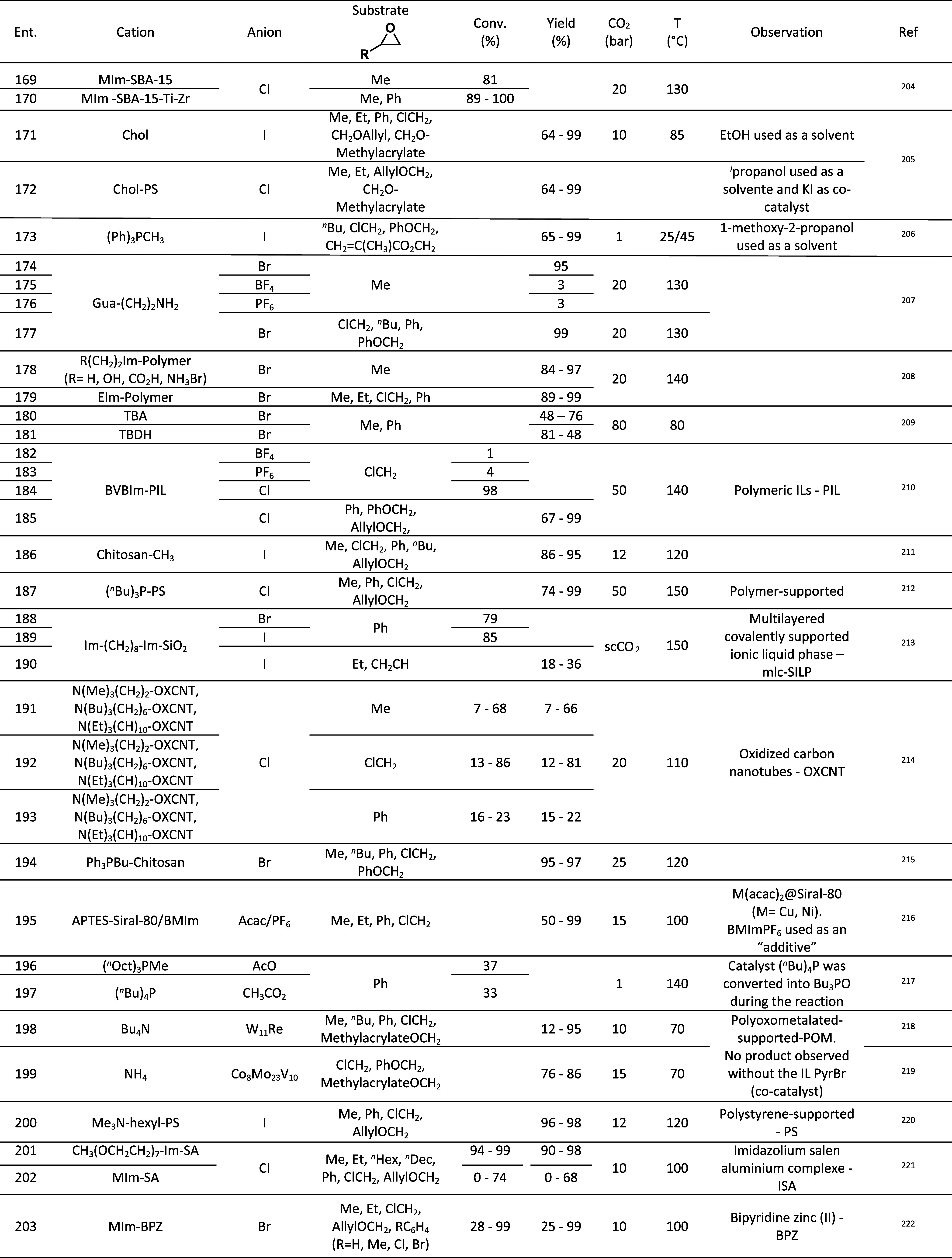

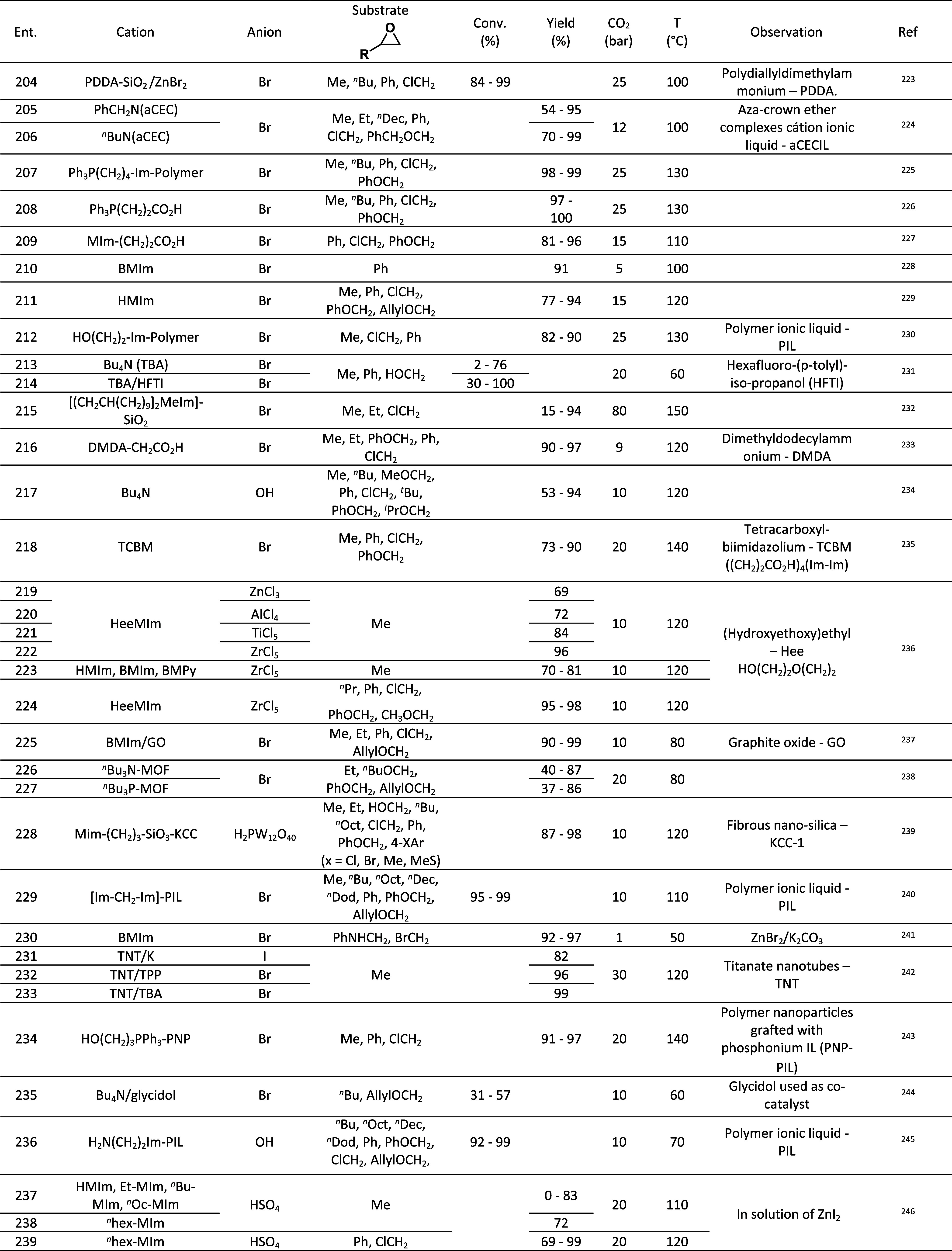

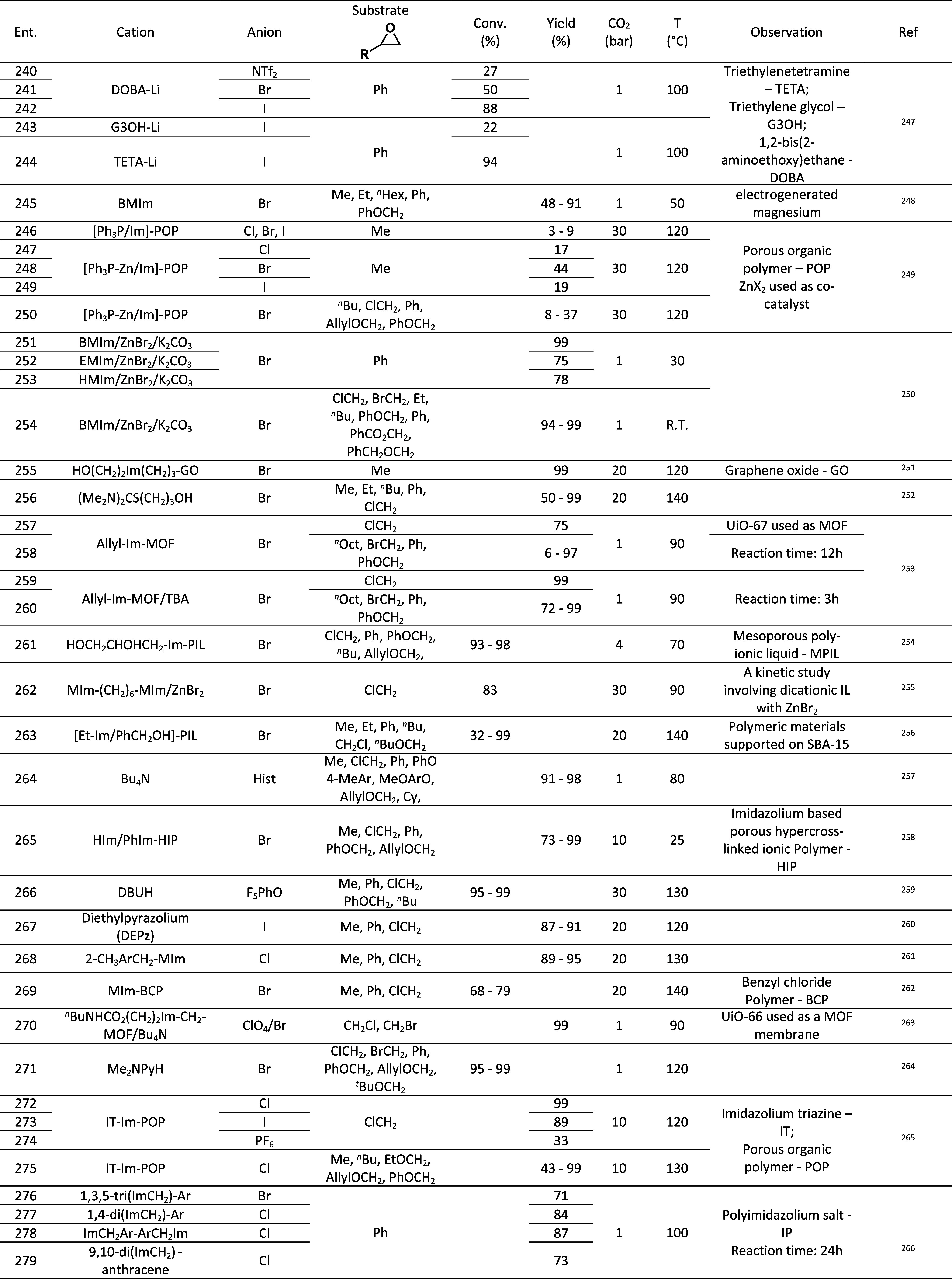

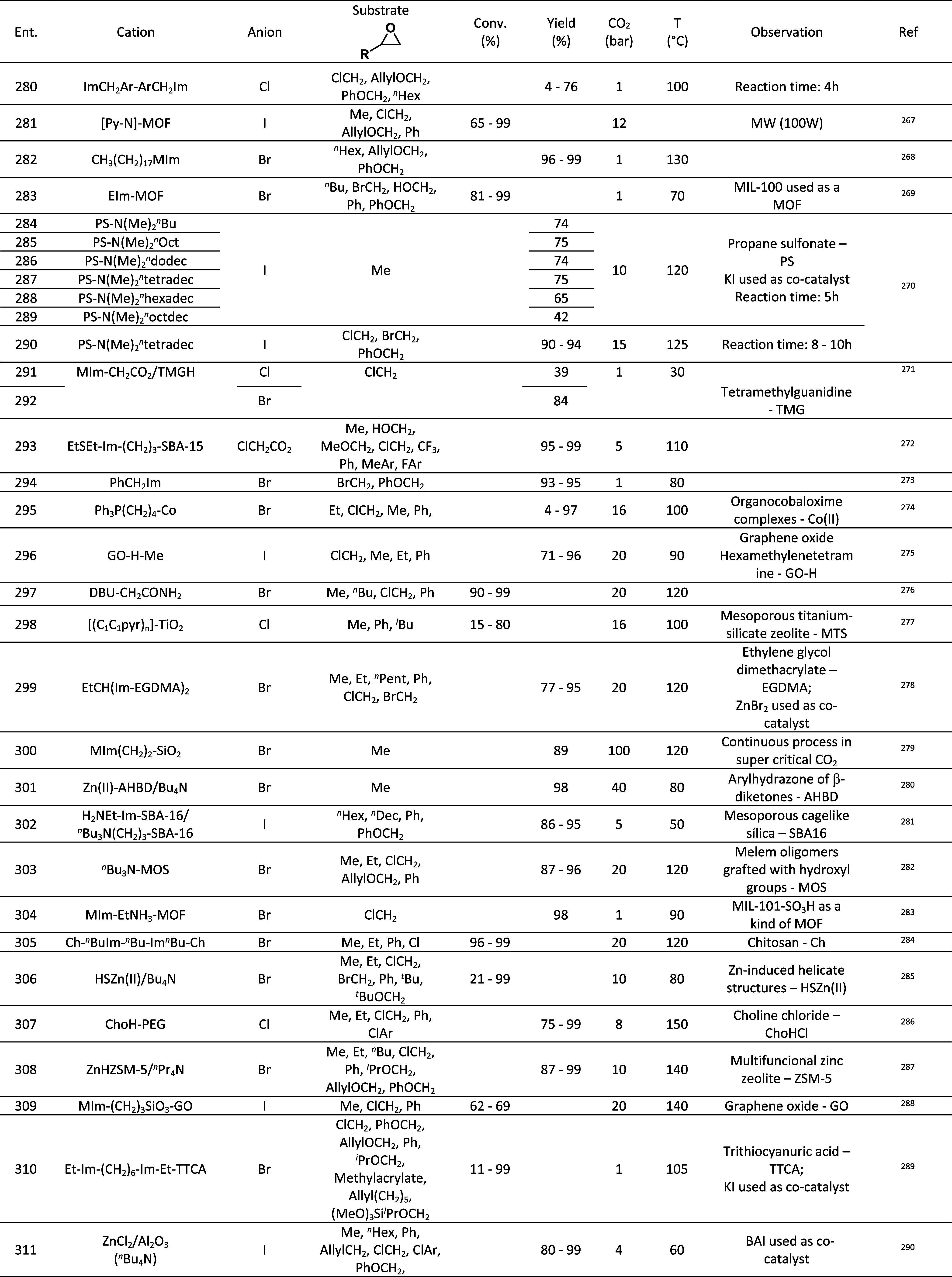

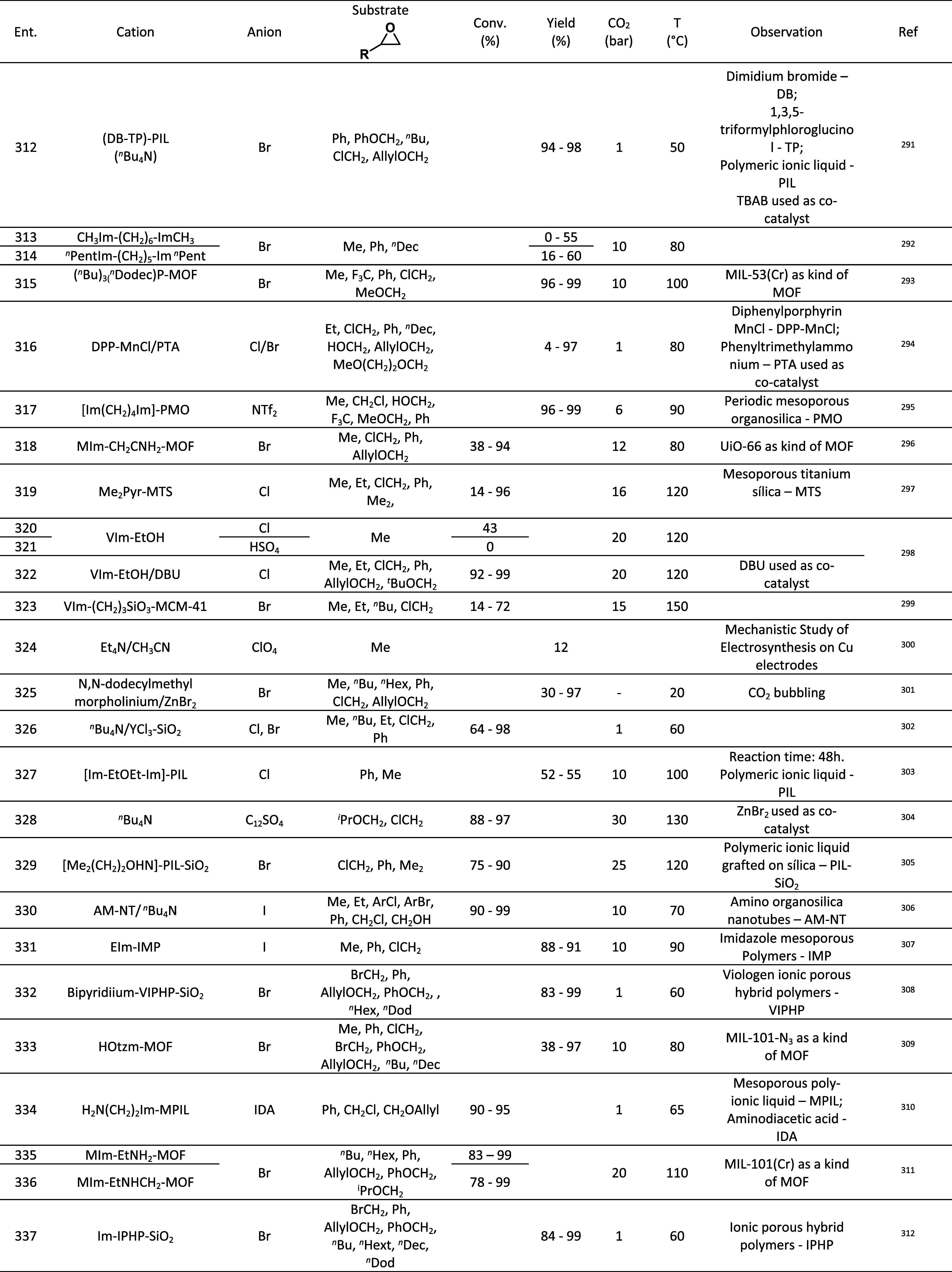

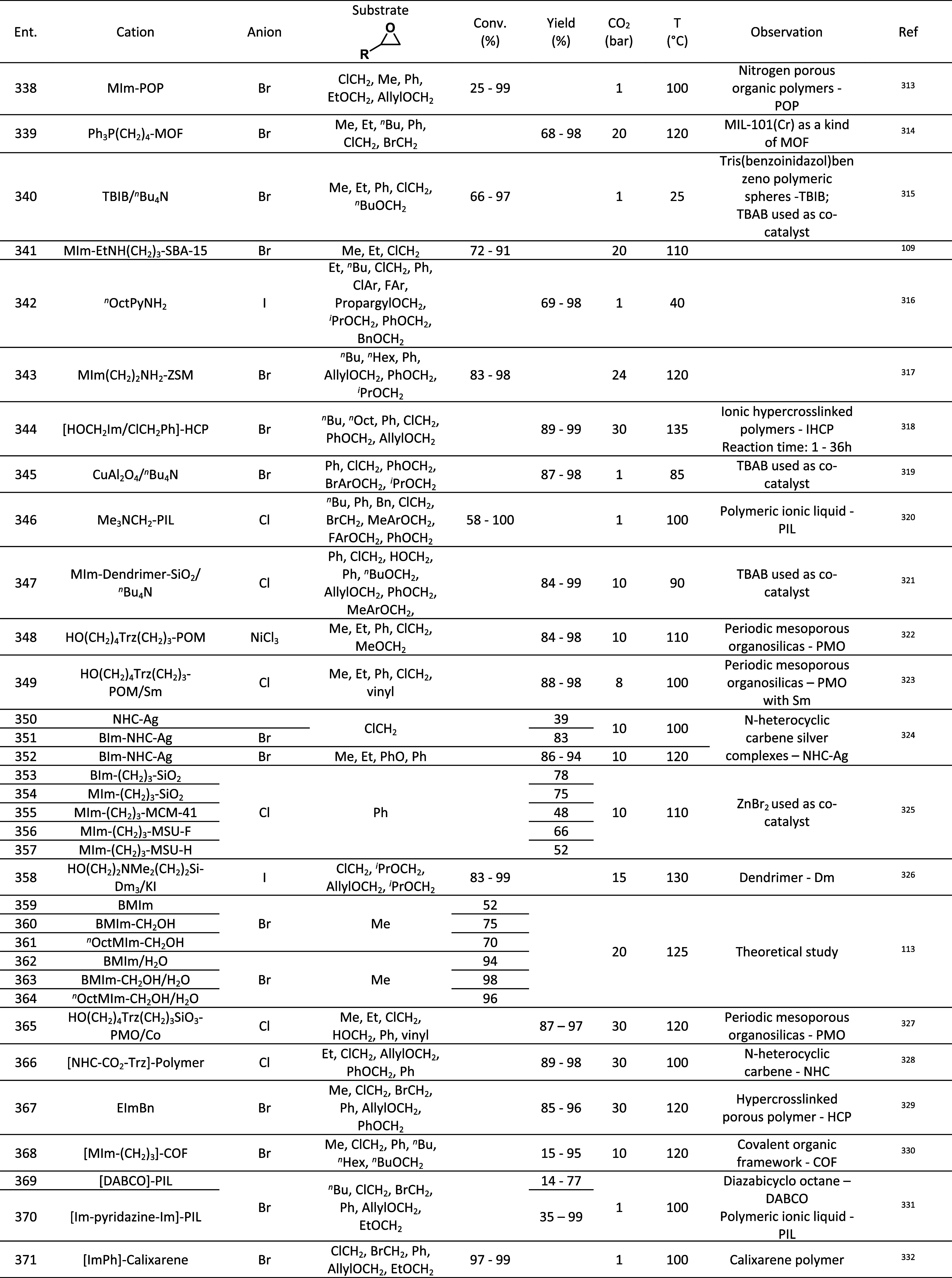

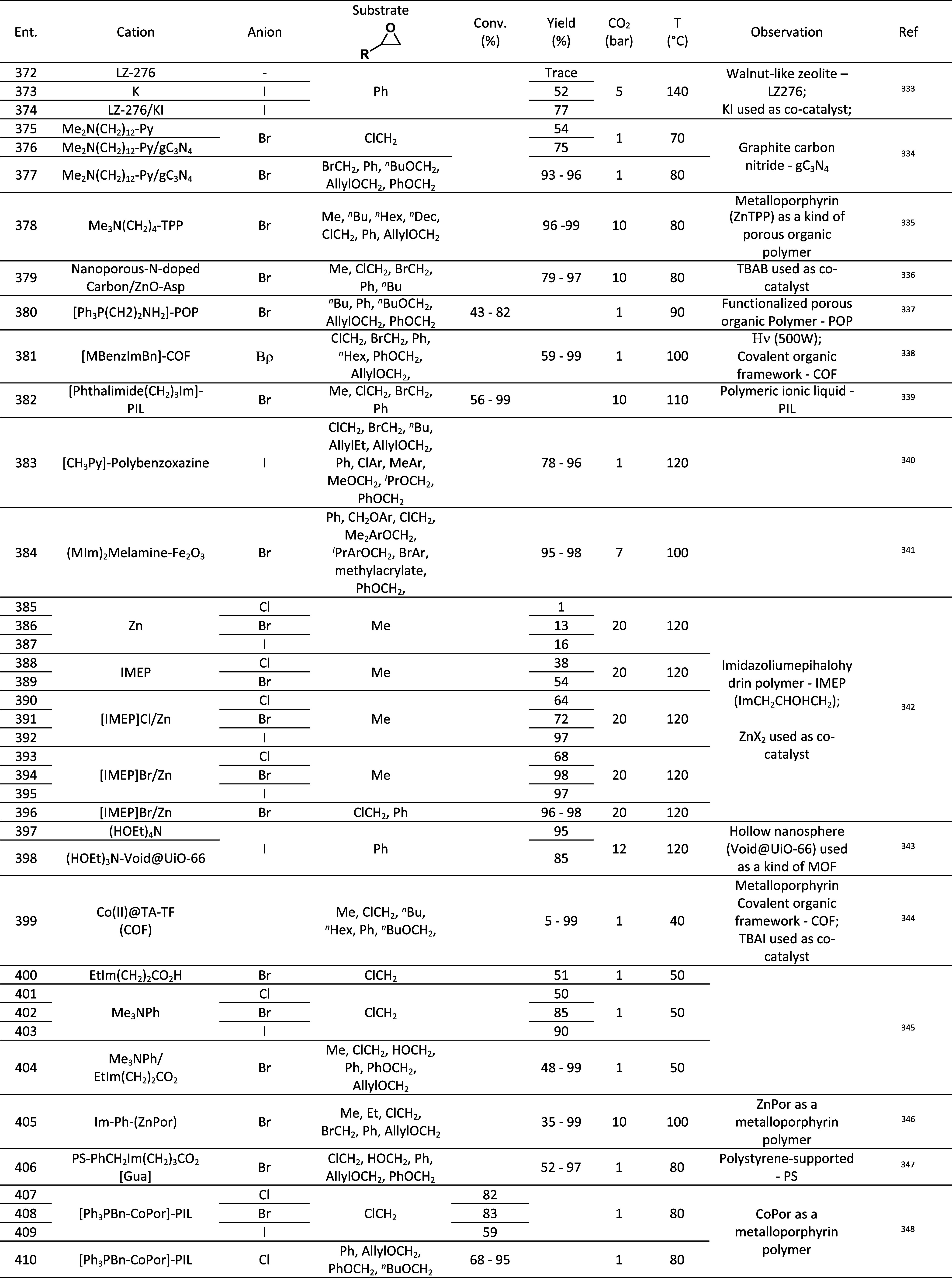

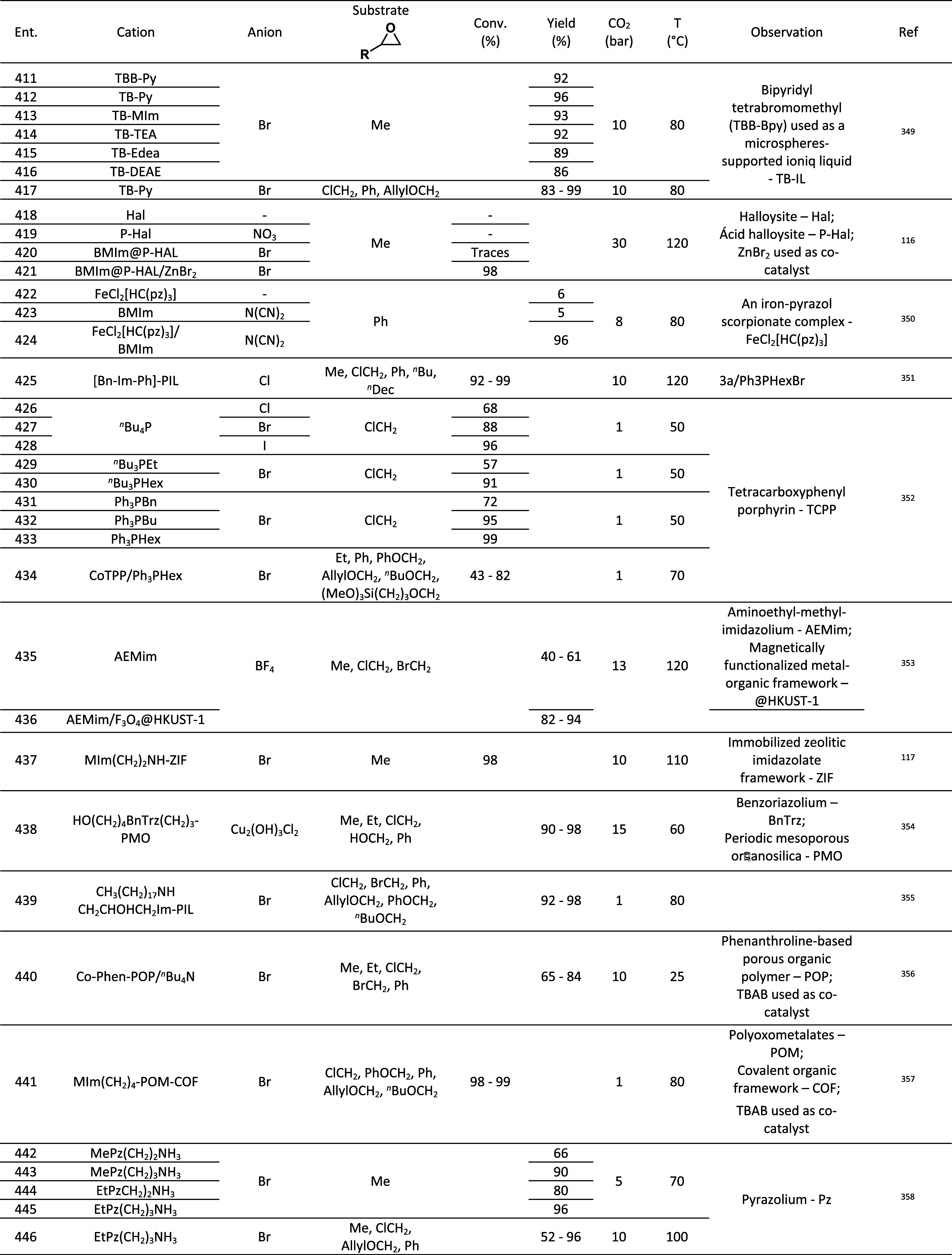

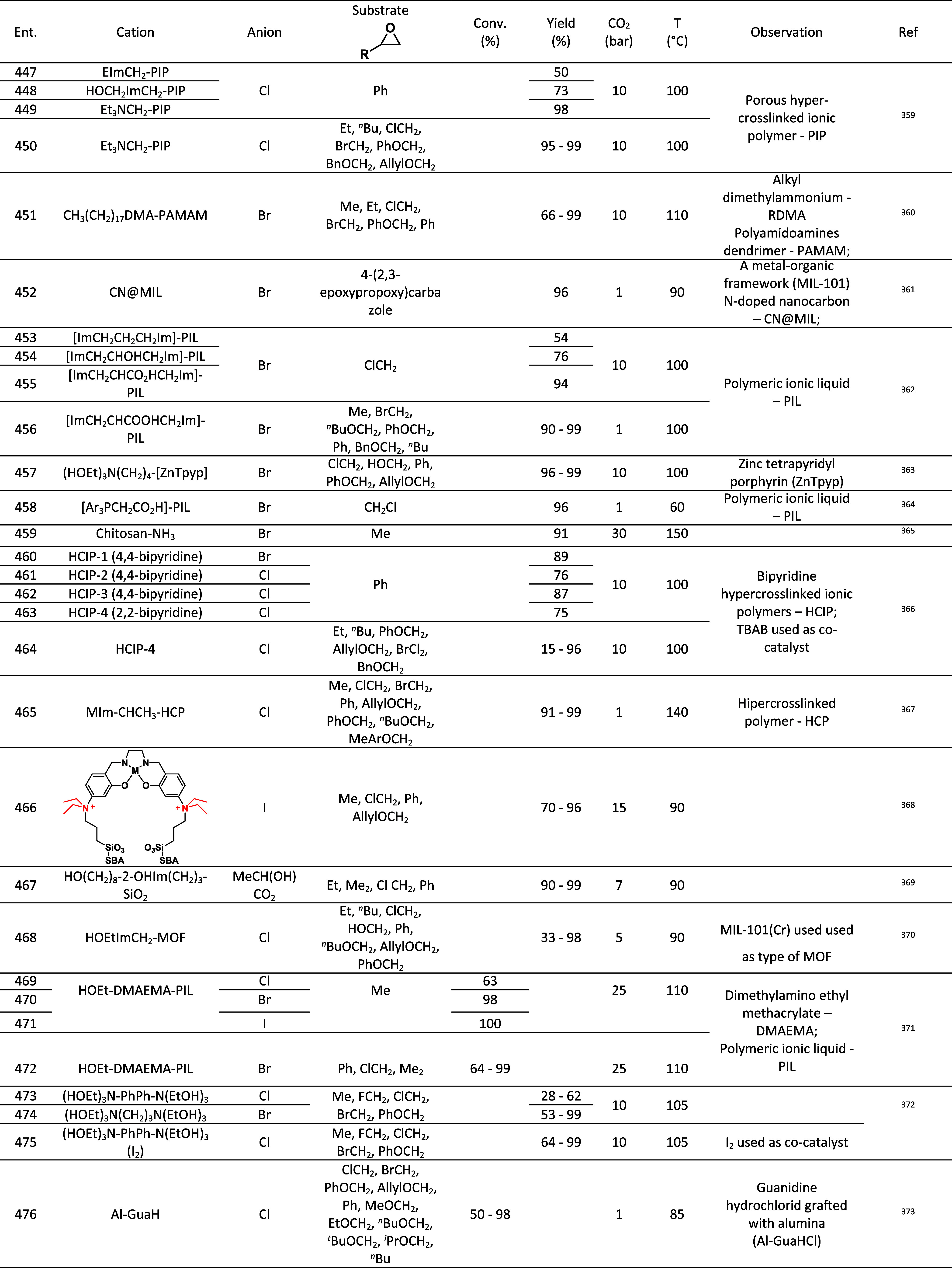

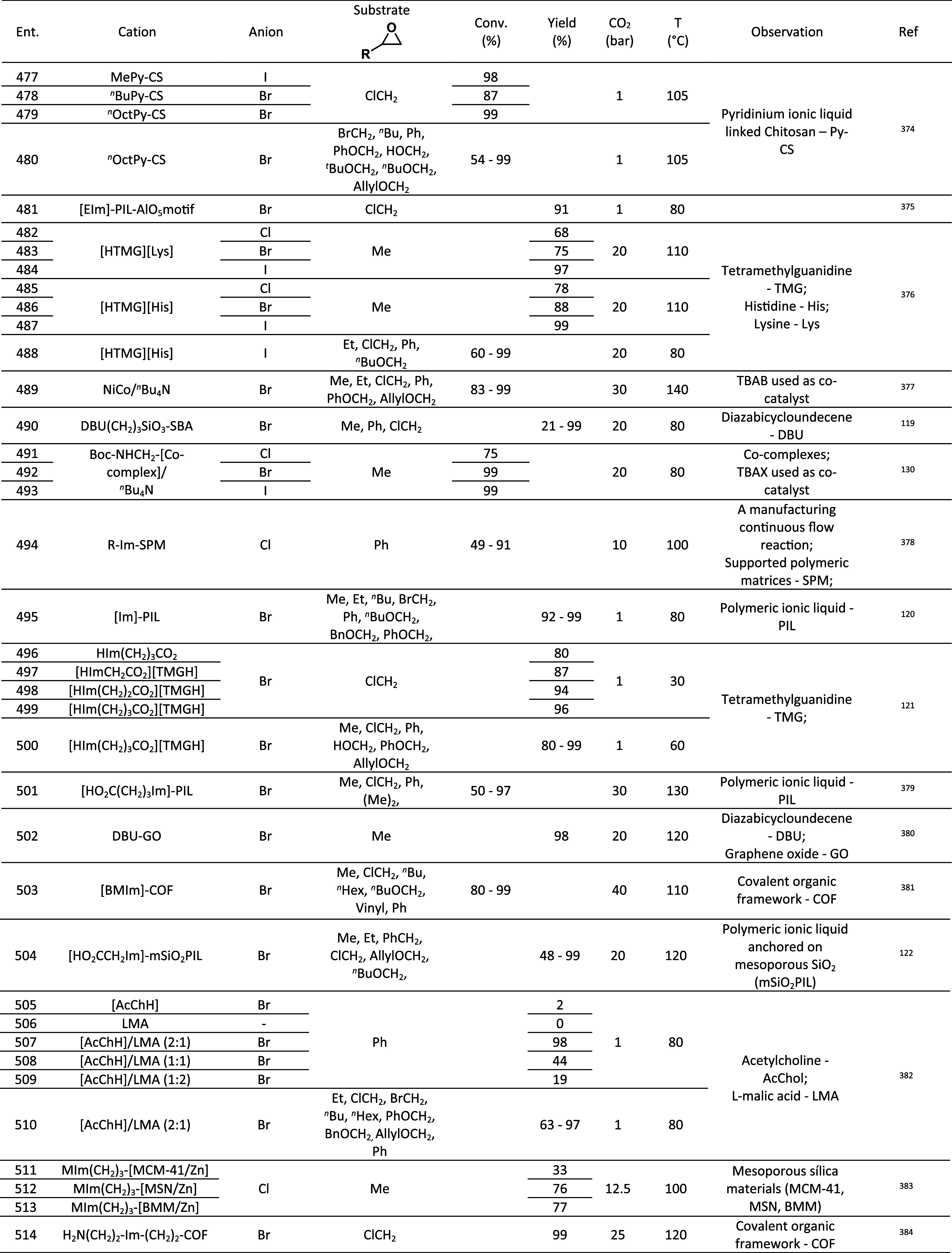

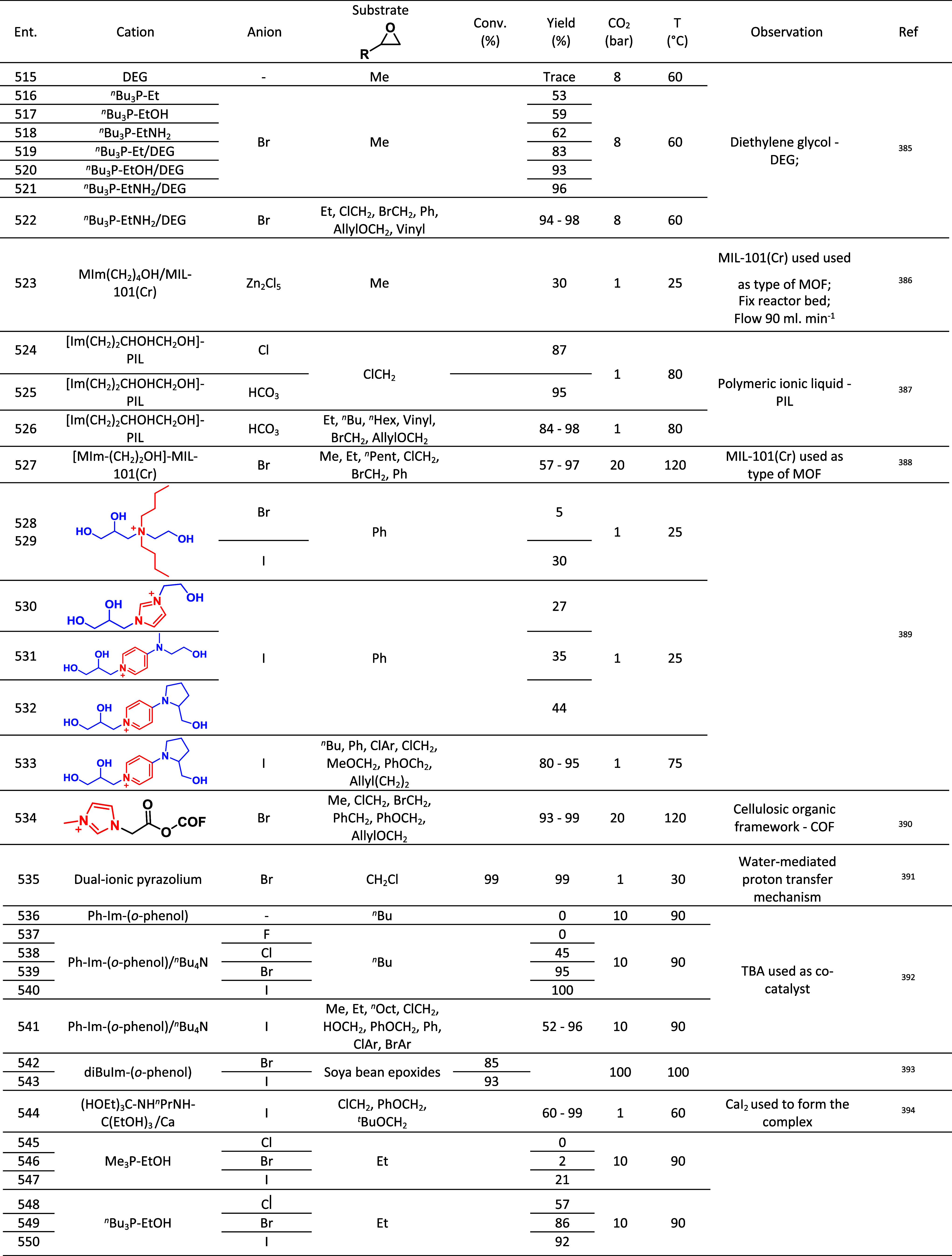

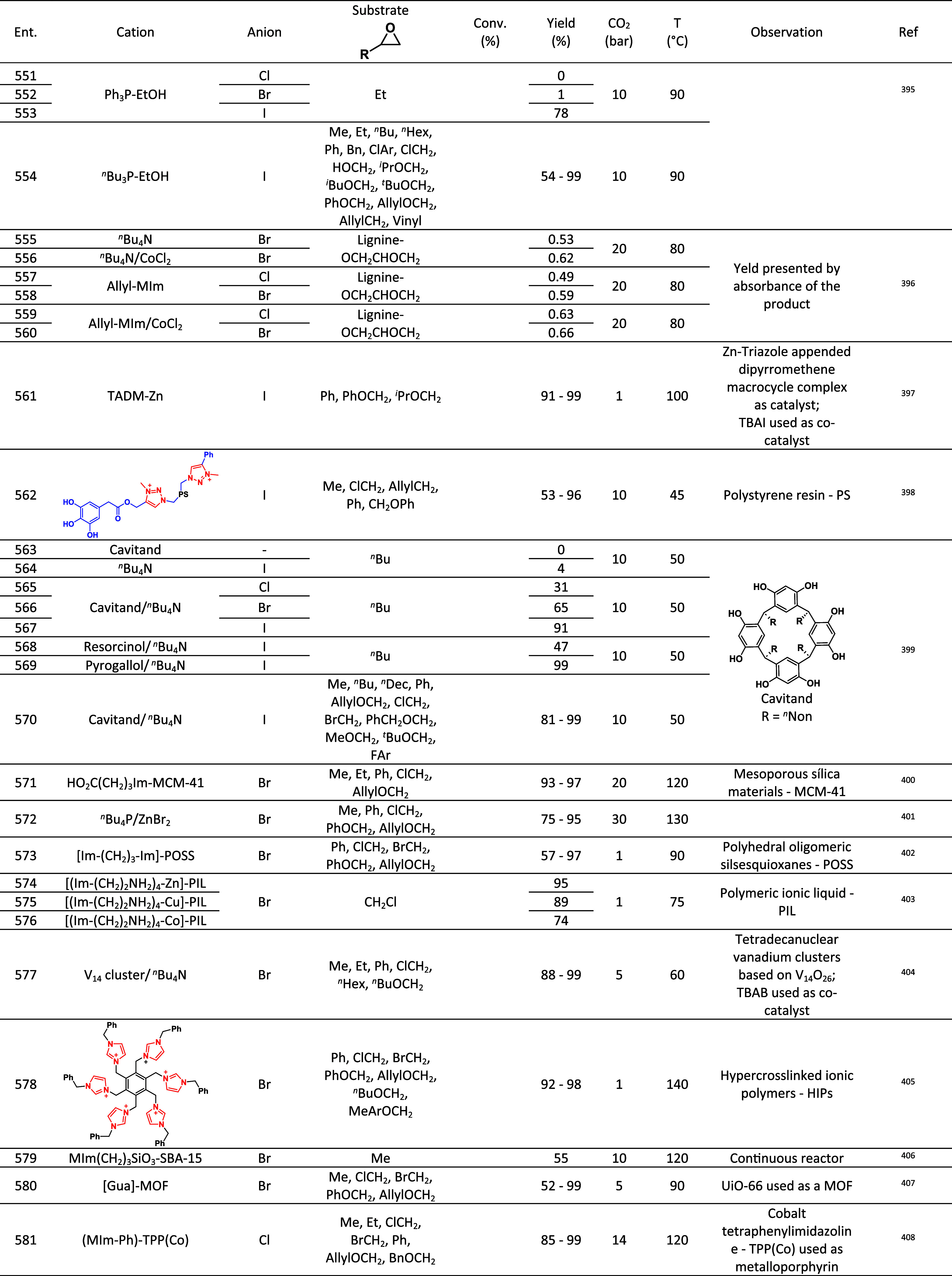

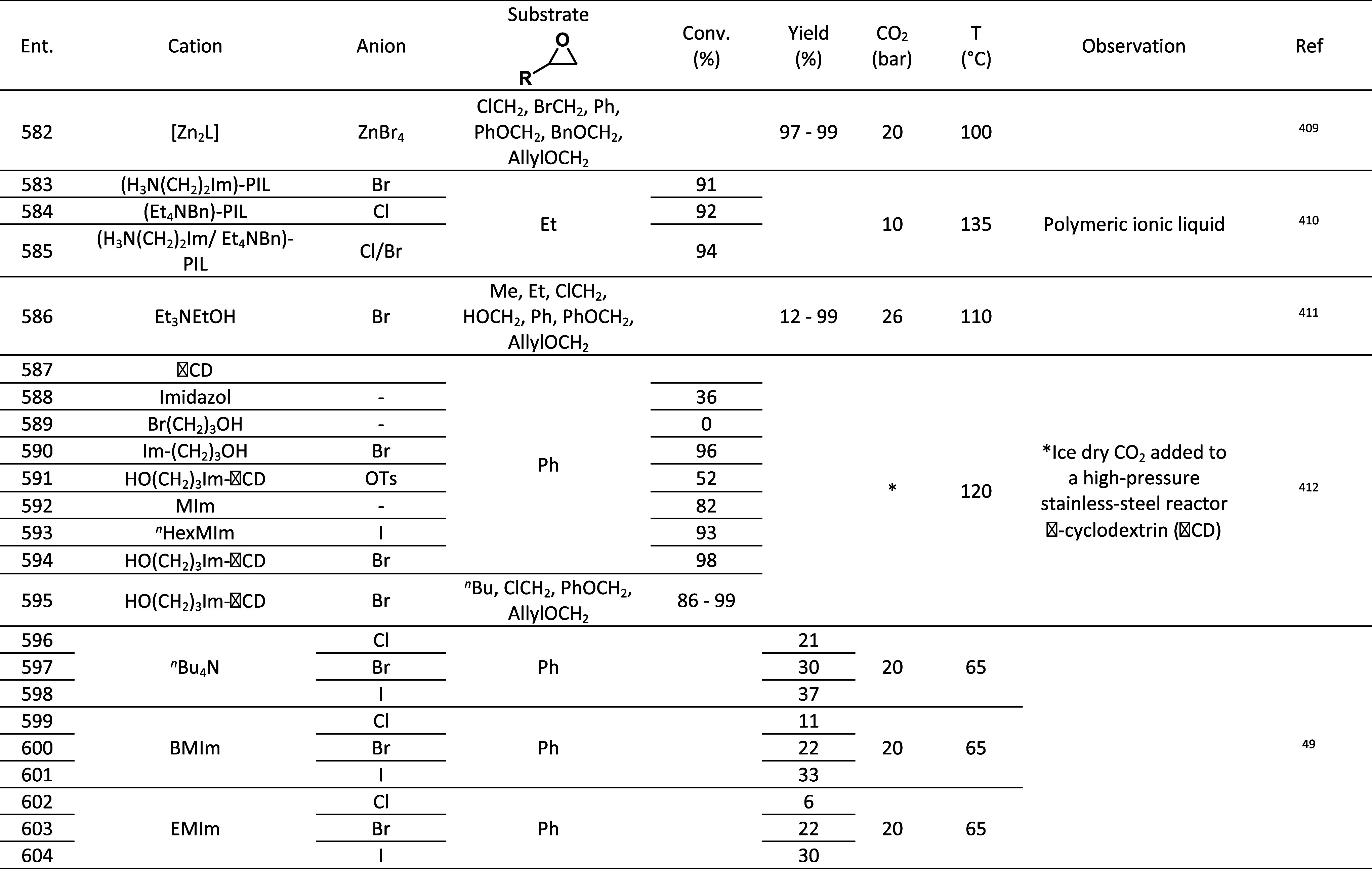
Carbonation of Monosubstituted Epoxides in the Presence of ILs

The rates of reactions, where the starting materials are charge-neutral and the formation of the activated complex involves the development of charges, are accelerated by using an ionic liquid solvent. This extends the Hughes-Ingold rule, initially devised for molecular solvents, to ILs, highlighting their high polarity. In cases where the formation of hydrogen bonds between a nucleophile and the anion of an ionic liquid is possible, the reaction is further accelerated. This can be controlled by enhancing the hydrogen-bond-acceptor ability of the anion, ensuring caution to prevent the anion from becoming nucleophilic enough to compete in the reaction. Formation of hydrogen bonds between the nucleophile and the cation will reduce its reactivity and should be avoided. In situations where there is no opportunity for the nucleophile to hydrogen-bond to either the anion or cation of the ionic liquid, other effects, such as the self-association of the ionic liquid, may become important. Amines, for instance, exhibit higher nucleophilicity in ILs compared to molecular solvents like dichloromethane and acetonitrile.^85^

Monosubstituted epoxides (Table 3), with only one substituent attached to the epoxide carbon atoms, experience less steric hindrance and offer a more accessible reactive site for nucleophilic attack. In addition, monosubstituted epoxides are generally more electrophilic than their disubstituted counterparts, rendering them more reactive toward nucleophiles. It is crucial to acknowledge that factors such as the choice of nucleophile, reaction conditions, and catalysts can influence the reactivity of epoxides. However, in general, disubstituted epoxides tend to pose greater challenges for carbonation compared to monosubstituted epoxides due to the steric hindrance and electronic effects associated with the presence of more than one substituent.

The carbonation of disubstituted epoxides (Table 4) is typically more challenging than that of ethylene glycol and monosubstituted epoxides due to steric hindrance and electronic effects. Disubstituted epoxides, having two substituents attached to the epoxide carbon atoms, introduce steric hindrance that can impede the approach of the nucleophile during the carbonation reaction. The presence of two bulky substituents limits the accessibility of the nucleophile to the reactive site, slowing down and complicating the reaction. Disubstituted epoxides can also exhibit electronic effects influencing the reactivity of the epoxide ring. The nature and positioning of the substituents impact the electron density distribution within the molecule. For instance, electron-withdrawing groups attached to the epoxide carbon atoms can destabilize the electrophilic character of the ring, making it less susceptible to nucleophilic attack and hindering carbonation reactions.

**Table 4:**
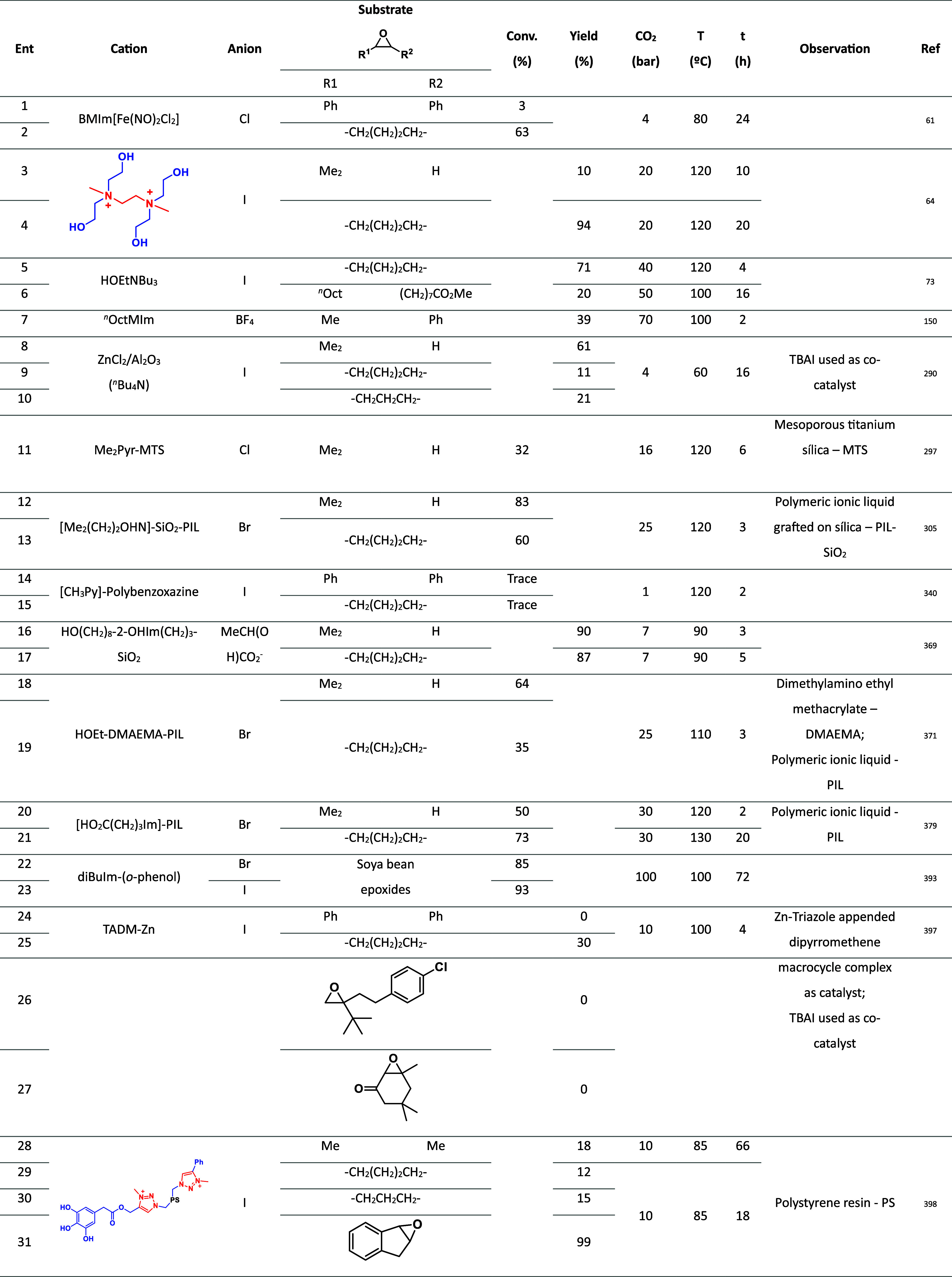

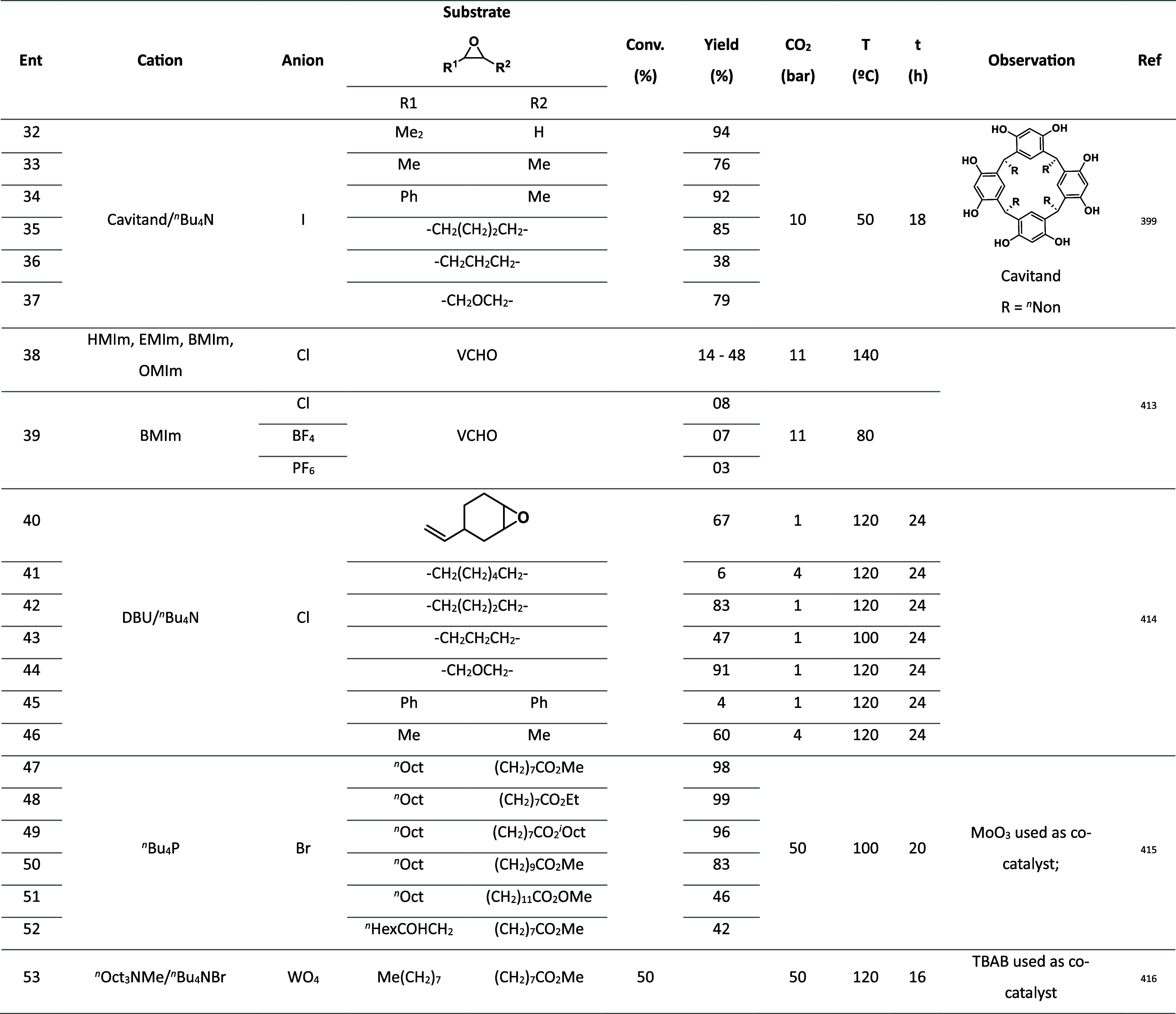
Carbonation of Disubstituted Epoxides in the Presence of ILs

The reactivity of the epoxide can be enhanced by employing Lewis acids, which, through interaction with the *O*-moiety, elevate the electrophilicity of the epoxide carbons. This is commonly achieved by using aluminum and aluminum-containing compounds. In some cases, hydrogen-bonding activation is assumed (see Scheme 5), predominantly employing *O*-substituted salts like simple 1-ethanol-3-methylimidazolium halides^86^ or polymeric ILs.^87^ The halide effect, in conjunction with hydrogen bonds, is also influential, and the utilization of ethylene glycol (polar protic solvent) as an additive further underscores this effect (Scheme 5).^77,88–91^ Table 3 compiles the majority of the reaction conditions employed to facilitate carbonation reactions from epoxides, including various features and roles of anions, cocatalysts, conditions, additives, etc.

**Scheme 5:**
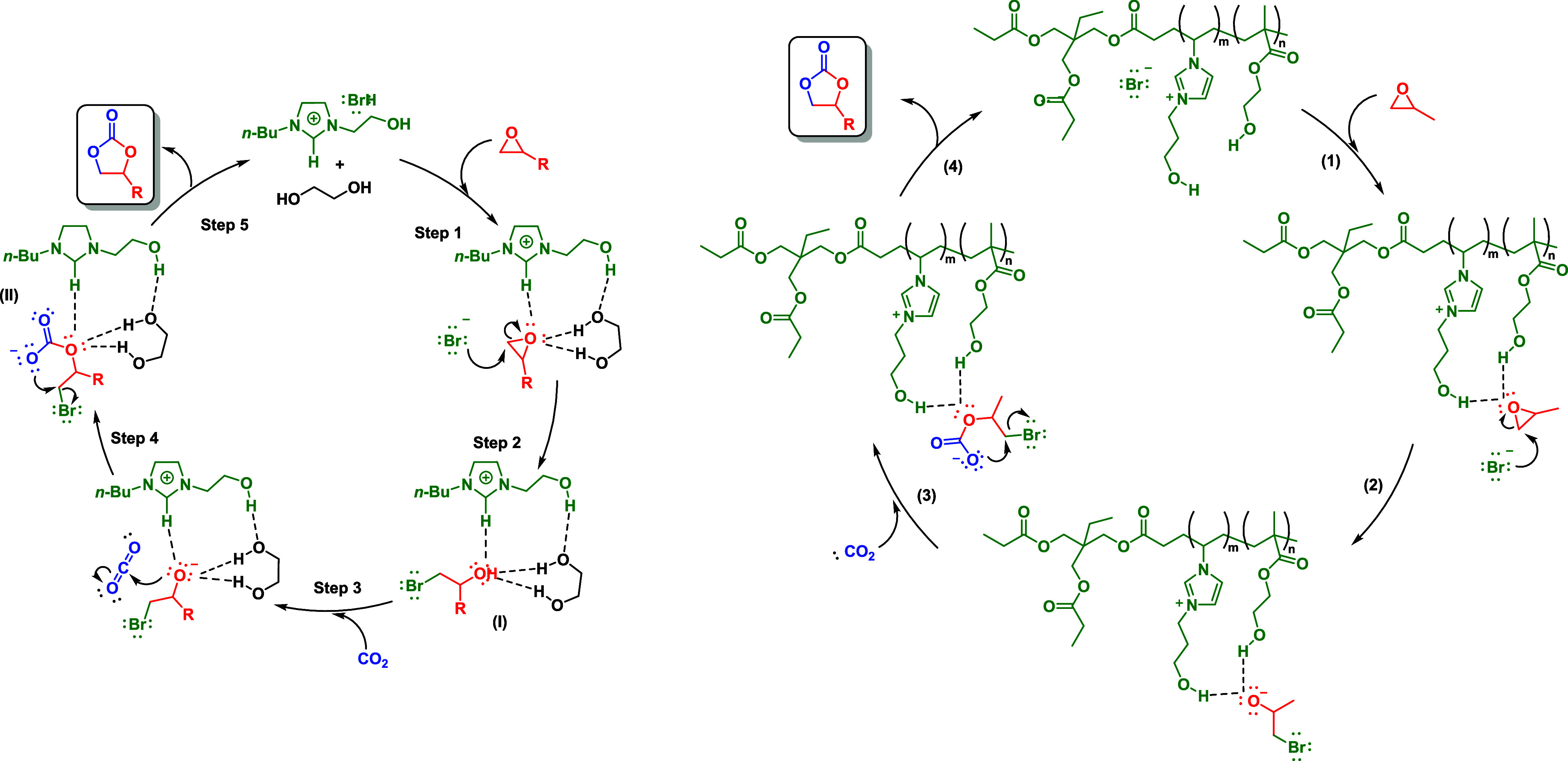
H-Bond Epoxide Activation in ILs (Left) and Poly-ILs (Right)[Fn s5fn1]

1 Glycidol derivatives facilitate the activation of epoxides due to the presence of H bonds.^86,87^

Another important aspect is the CO_2_ solubility in the ILs. There are many factors capable of altering the solubility of a chemical species. Although this may be one of the most neglected aspects when considering carbonation reactions in ILs, a few words are worth noting. For carbonation reactions, it has to be considered not only the solubility of CO_2_ in the reaction medium but also the solubility of the catalytic system (homogeneity or heterogeneity) and reactants, as discussed elsewhere.^18^ Additionally, the pressure and temperature are known to directly influence the CO_2_ solubility in ILs. Therefore, it is not uncommon for carbonation reactions to be conducted under high-pressure and high-temperature conditions in these ionic fluids.

A groundbreaking study has investigated several strategies to enhance CO_2_ solubility in ILs.^92^ The presence of CO_2_ can promote the increased solubility of other gases with poor solubility in the IL phase, necessitating consideration in carbonation reactions. Certain structural attributes, such as flexible alkyl chains to augment the free volume, have been recognized to enhance the CO_2_ solubility in various compounds, including polymers and ILs. Notably, features such as carbonyl groups and long alkyl chains with branching or ether linkages contribute to this enhancement. Despite these considerations, stability and viscosity remain crucial properties for practical applications. Another important factor to consider is the choice of anion, which significantly influences CO_2_ solubility in ILs, particularly in nonfluorous ILs where fluorination often enhances solubility. However, fluorinated ILs may pose environmental concerns compared to nonfluorinated alternatives. In this context, anions serve not only as bases or nucleophiles (or neither) but also as key determinants of CO_2_ solubility, thereby influencing reaction performance.

It is a demonstrated fact that conventional ILs are not particularly effective at dissolving CO_2_.^93^ The solubility of this gas occurs within the ’pores’ (nanostructures) of the liquid, so strategies aimed at enhancing solubility typically yield positive results. One should remember that the chemical sorption of CO_2_ is dependent on the IL structure, which differs from the simple physical sorption.

In another investigation, researchers conducted molecular dynamics (MD) simulations on ten distinct ILs, both in their pure forms and when saturated with CO_2_, which revealed a few insights.^94^ The simulations suggested that the absorption of CO_2_ was facilitated by weak interactions between cations and anions. To enhance the physical absorption of CO_2_, it was proposed that larger ions should be used to decrease the overall ion density. The study recommended that cations should be significantly larger than anions to create regions with repulsive cations in close proximity and cautioned against functionalizations that promote attraction between different cation groups.

The utilization of machine learning techniques for predicting the solubility of CO_2_ in diverse types of ILs across a range of temperatures and pressures is noteworthy. It is imperative to acknowledge that experimental solubility data, particularly in instances of very low solubilities, may exhibit considerable uncertainties. Leveraging the data set provided by the 10.116 experimental measurements of CO_2_ solubilities in ILs, however, allowed for reliable predictions concerning the solubility of this gas in ILs.^95^

## Mechanistic and Theoretical Investigations

Mechanistic^96–99^ and theoretical^100–123^ investigations enabled an in-depth understanding of the importance of ILs in this transformation and their major roles. In situ high-pressure attenuated total reflection infrared (ATR-IR) spectroscopy was used to study the molecular interactions between dissolved CO_2_ and different imidazolium-based room-temperature ILs. The results showed that the cation and anion species of the ILs affect the molecular state of dissolved CO_2_ due to the Lewis acid–base interaction between the IL anion species and CO_2_. The spectroscopy was also applied to the [C_1_C_4_]­[BF_4_]-propylene oxide-CO_2_ system to evaluate the reactivity of [BF_4_–CO_2_]^−^ and propose a new plausible catalytic cycle in which the [BF_4_–CO_2_]^−^ species first attacks the electrophilic epoxide carbon, followed by cyclization of the corresponding intermediate to give a propylene carbonate product as well as [C_1_C_4_]­[BF_4_]. The studies also revealed that the choice of the cation species of the IL can tune the Lewis acid–base interaction between BF_4_ and dissolved CO_2_. Additionally, it was found that the strong Lewis acid–base interaction between the ILs and the dissolved CO_2_ has no promotional effect on the solubility of CO_2_.^124^

There are several theoretical calculations on the reaction mechanism for the carbonation of epoxides. However, in most of the cases, it is assumed that the nucleophile (halide or onium) is present in the media and no consistent studies on the ion solvation or organic salt ionicity has yet been reported. However, it is worth to mention that the mechanisms of reductive functionalization of CO_2_ to formamide catalyzed by *N*-heterocyclic carbene (NHC) were comprehensively studied with DFT calculations.^125^

The cycloaddition of CO_2_ onto propylene oxide catalyzed by ammonium and guanidinium salts has been investigated by using DFT to understand the catalytic mechanism and the role of the cation and the anion. Two different possible pathways were considered, but it has been found that the one whereby the activation of the epoxide by the onium salt occurs before the addition of CO_2_ was consistent with the experimental findings. The rate-determining step was found to be the ring opening of the epoxide that results from the nucleophilic attack of the anion of the catalyst on the nonsubstituted carbon of the epoxide ring. It was also found that in order to have an efficient catalyst, it is necessary to have an anion with an ambivalent nature (a high nucleophilicity and good leaving group ability).^126^

The mechanism of insertion of CO_2_ into styrene oxide (STYO), in both the absence and presence of the catalyst C_1_C_4_.Br, was investigated through calculations based on density functional theory at the B97X-D level. Two different routes were considered, and it was shown that they are energetically accessible and compete with each other. For both routes, the rate-determining step is the ring opening of STYO, resulting from the nucleophilic attack of Br^–^ on the less hindered carbon atom of STYO, which is mainly associated with participation of the cation and the anion from the catalyst in the reaction. Reactive indices and noncovalent interaction analysis were used as tools to investigate this mechanism. This work provides a better understanding of the underlying mechanism, and the supplied data offer valuable support for the design of more efficient IL catalysts.^127^

The combination of Ch (choline chloride) and ZnBr_2_ generates three new stable complexes: [Ch]_2_[ZnBr_2_Cl_2_], Ch+ZnBr_2_Cl, and Ch+ZnBrCl_2_. The latter two are derived from the dissociation of [Ch]_2_[ZnBr_2_Cl_2_]. The detailed mechanism of a coupling reaction catalyzed by the more stable complex, Ch+ZnBrCl_2_, is explored. It has been elucidated that the attack from the Zn complex and the Br anion is the major factor in promoting the cleavage of the C–O bond of PO. Finally, the performance of [Ch]_2_[ZnBr_2_Cl_2_] is also investigated, showing lower activity and indicating that it should dissociate to achieve a better catalytic effect.^110^

Seven reaction pathways were identified, showcasing two- or three-step mechanisms involving epoxide ring opening, CO_2_ insertion, and cyclic carbonate formation (Scheme 6). The study highlighted the importance of the hydroxyl group in HEMIMC (1-(2-hydroxyl-ethyl)-3-methylimidazolium chloride), which facilitates hydrogen bonding and stabilizes reaction intermediates. The catalytic mechanism emphasizes the role of the Cl^–^ anion in promoting nucleophilic attack, while the hydroxyl group enhances the stabilization of the key intermediates. DFT calculations also revealed that the incorporation of the hydroxyl group significantly lowers the reaction energy barriers compared to nonhydroxylated analogs, supporting the high catalytic efficiency of HEMIMC in CO_2_ fixation.^128^

**Scheme 6:**
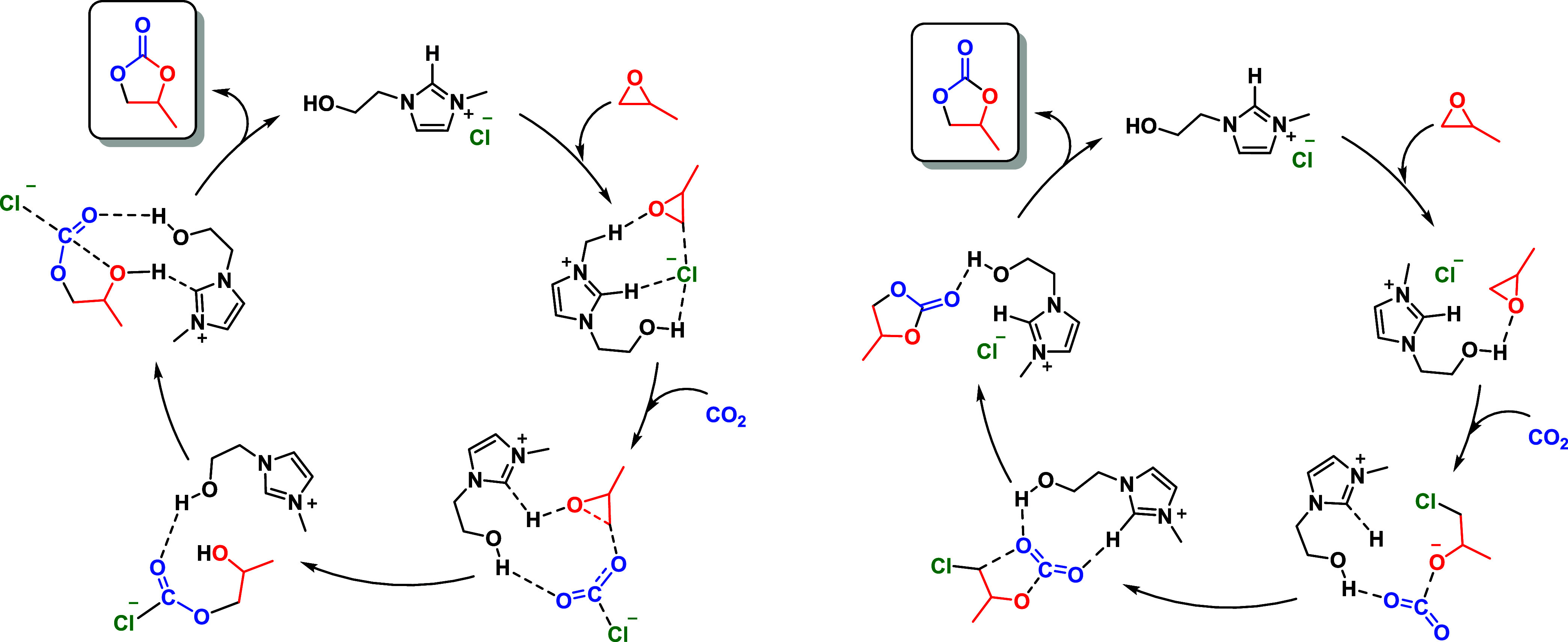
Two- and Three-Step Carbonation Reactions in ILs Based on DFT Calculations

## Diversity of Carbonation Reactions in ILs Systems from Epoxides

Polycarbonates have also been synthesized using ILs.^129,130^ ILs, such as imidazolium-based salts, serve as efficient catalysts in the copolymerization of CO_2_ with epoxides. This copolymerization leads to the formation of polycarbonates with high carbonate content and low polydispersity. The structure of the IL significantly affects the reaction’s outcome, with variations in the alkyl chains and anions influencing catalytic activity, product yield, and selectivity. ILs were also applied as chain transfer agents in the one-pot synthesis of CO_2_-based polycarbonates. The anion in the IL played a crucial role in the reaction mechanism, with nucleophilic anions, such as chloride, acting as cocatalysts, while non-nucleophilic anions, such as BF_4_^–^, facilitate the chain transfer process. This process enabled the formation of ion-containing polycarbonates through immortal copolymerization of propylene oxide and CO_2_. The findings highlight that the structure of the IL influences both the polymerization process and the resulting polycarbonate’s properties, providing insights into optimizing polycarbonate synthesis with ILs.

One-pot synthesis of dimethyl carbonate from methanol, propylene oxide, and carbon dioxide has been achieved using supported choline hydroxide/MgO catalysts,^131^ metal oxides,^132^ in 1-alkyl-3-methyl imidazolium salts with varying alkyl groups (C-2, C-4, C-6, C-8) and anions (Cl^–^, Br^–^, BF_4_^–^, PF_6_^–^),^133^ or using imidazolium halides immobilized on mesoporous amorphous silica^134,135^ or dicationic quaternary ammonium salts.^136^

The copolymerization of phenyl glycidyl ether (PGE) and CO_2_ was performed in the presence of IL catalysts. C_1_C_4_ chloride, tetrabutylammonium chloride, and 1-*N*-butylpyridinium chloride were used as catalysts for this reaction, which was carried out in a batch reactor. All of the IL catalysts demonstrated good catalytic activity for the synthesis of polycarbonates with very low polydispersity, close to 1. The carbonate content, TON, and average molecular weight of the copolymer were influenced by the structure of the IL. High CO_2_ pressure enhanced both TON and carbonate content due to the increased CO_2_ absorption in the PGE solution. ZnBr_2_ and a Zn–Co cyanide complex were also tested as catalysts and/or cocatalysts for this reaction to compare their catalytic performance with the imidazolium salt ILs.^137^

The synthesis of high surface area PILs for the catalytic conversion of CO_2_ to ethylene carbonate was disclosed. Designed PILs with various anions and alkyl chain lengths to explore their efficiency in CO_2_ cycloaddition reactions were demonstrated. The porous structure of the PILs enabled a better diffusion of reactants, which improved the catalytic activity under mild conditions. Through in situ FTIR spectroscopy, the relationship between PIL structure and catalytic performance was elucidated, highlighting the significance of optimizing surface area and ion structure for enhanced CO_2_ utilization.^138^

The kinetic aspects of styrene carbonate synthesis from styrene oxide and carbon dioxide using a continuous flow tube-in-tube gas–liquid reactor were disclosed. The study utilized a combination of tetrabutylammonium bromide (TBAB) and ZnBr_2_ as a binary homogeneous catalyst system, which significantly enhanced the catalytic performance. The continuous flow system allowed for rapid CO_2_ permeation through a Teflon AF-2400 membrane, leading to 100% conversion of styrene oxide within a short reaction time of 45 min. Key parameters, such as reaction temperature, CO_2_ pressure, and residence time, were systematically varied to optimize reaction conditions. The kinetic analysis showed a first-order dependence on styrene oxide, CO_2_, and TBAB concentrations, whereas ZnBr_2_ exhibited a nonfirst-order behavior. The study highlighted the advantages of flow chemistry, particularly in achieving higher reaction rates, enhanced mass transfer, and improved scalability compared to batch processes.^139^ A few other articles describe similar performances and conclusions.^138–141^

Some have also used a continuous flow system for the synthesis of CO_2_-based polycarbonates, focusing on improving the reaction efficiency and control. The flow system allowed for better management of reaction parameters, such as temperature and pressure, leading to more consistent and reproducible results. The continuous flow method demonstrated superior scalability and process control compared to batch processes, making it a promising approach for industrial polycarbonate production.^140^ ILs in this synthesis played a crucial role as catalysts or cocatalysts in the copolymerization of CO_2_ with epoxides. ILs facilitate the formation of polycarbonates, affecting key factors such as the carbonate content and molecular weight of the final product. The structure of the IL, particularly the type of anion and the alkyl chain length, significantly influenced the reaction’s efficiency and selectivity. In some cases, ILs promoted nucleophilic attack by activating CO_2_, making them critical for improving the overall reaction performance.

The synthesis of cyclic carbonate by the cycloaddition reaction of CO_2_ and epoxide, catalyzed by imidazolium salt ILs, was carried out under supercritical CO_2_ conditions. To elucidate the effect of imidazolium-based ILs on reaction kinetics, the cycloaddition reaction with various ILs, featuring different cations and anions, ranging from C-2 to C-6 alkyl chains and BF_4_^–^ and Br^–^, respectively, was studied at various temperatures (90–130 °C). Based on the batch experiment results, it was observed that ILs with longer alkyl chain cations and BF_4_^–^ anions, specifically 1-hexyl-3-methylimidazolium tetrafluoroborate, exhibited comparatively higher catalytic activity. Pseudo-first-order reaction kinetics predominated during cyclic carbonate synthesis under supercritical conditions. The kinetic analysis of the cycloaddition reaction in this study confirmed that maximum conversion efficiency and minimum activation energy (37 kJ/mol) were associated with Hmim.BF_4_, making it a promising catalyst for cyclic carbonate synthesis under supercritical conditions.^142^

The catalytic asymmetric cycloaddition of CO_2_ to epoxides using chiral bifunctional ILs was investigated. The ILs, consisting of an imidazolium cation and various axial anions, were designed to promote enantioselective carbonations. The study highlighted the influence of both the cation structure and the anions on the catalytic activity and enantioselectivity, with specific combinations showing higher yields of chiral cyclic carbonates. The ILs facilitated the reaction without producing polycarbonates or other byproducts. The order of catalytic activity was OAc^–^ > CF_3_CO_2_^–^ > CCl_3_CO_2_^–^ > OTs^–^, and the enantioselectivity was found to improve with OTs– over the others, showcasing the crucial role of ILs in the asymmetric transformation process.^143^

A tandem^144^ oxidation-carbonation reaction was disclosed. The article explored a one-pot tandem synthesis of styrene carbonate starting from styrene and CO_2_, using a nanogold-IL catalyst system. In this reaction, the nanogold catalyst supported on multiwalled carbon nanotubes (Au/CNT) facilitated the epoxidation of styrene (oxidation), whereas the C_1_C_4_.Br-ZnBr_2_ was crucial for the cycloaddition of CO_2_ to the in situ-formed styrene oxide. The reaction proceeded in two steps, with the IL catalyst promoting the cycloaddition phase under supercritical CO_2_ conditions. The designed catalyst led to a significant overall yield of 60%. The study demonstrated the advantages of this approach in improving the reaction efficiency and simplifying the synthesis process by avoiding intermediate isolation.

## Outlook and Future Perspectives

The synthesis of cyclic carbonates via the carbonation of epoxides, catalyzed by IL systems, depends heavily on the nucleophilic and basic characteristics of the catalyst. The nucleophilicity of the anionic species plays a central role in promoting the cycloaddition reaction, while the base strength contributes to the activation of CO_2_ and epoxides. The efficiency of these reactions is also influenced by the leaving group ability, where weaker leaving groups tend to impede the reaction’s progress. Water content, often overlooked, is another crucial factor, as it impacts the solvation and ionization of the catalyst. Residual water in ILs can facilitate the solvation and subsequent nucleophilicity of halide anions, especially in hydrophilic environments.

The solubility and ionicity of ILs are also paramount in determining their effectiveness in cyclic carbonate synthesis. Ionic liquids with higher ionicity promote better solubility in the reaction medium, thus, enhancing catalyst performance. However, despite the significant number of published works, several aspects remain unclear, and the current knowledge is still fragmented. A deeper understanding of the complex interplay among solubility, ionicity, nucleophilicity, and water content is needed. The reaction mechanism and the specific interactions between epoxides, CO_2_, and ILs remain complex, and more detailed theoretical and experimental investigations are required to clarify these points.

Future studies should prioritize the development of standardized kinetic protocols with strict control over water content and other critical parameters to allow meaningful comparisons between different catalytic systems. Standardized studies focusing on kinetic parameters such as the rate constants, turnover frequency (TOF), turnover number (TON), and induction periods are essential. Systematic investigations that independently vary the ionicity and solubility are also needed to clarify their respective roles in catalyst performance.

Given the significant influence of residual water on anion solvation and catalytic activity, future studies should report the exact water content in ILs or organic salts. Investigating the quantitative effect of water on induction periods and overall catalytic performance is highly recommended, although this is a challenging task and is not always feasible. In situ spectroscopic techniques, such as ATR-IR, Raman, or ESI-MS­(/MS), can provide real-time monitoring of the ionic speciation, solvation dynamics, and nucleophile availability during the reaction. Additionally, advanced theoretical modeling, combining DFT and molecular dynamics simulations, can provide more realistic insights into ion dissociation, nucleophile reactivity, and CO_2_ activation pathways under actual reaction conditions.

The design of new catalyst structures with tailored anion–cation interactions and tunable ionicities represents another promising direction. This approach may offer improved control over reactivity, especially when targeting more challenging substrates such as disubstituted epoxides. Benchmark studies comparing different catalytic systems under strictly identical conditions, including the same substrate, CO_2_ pressure, temperature, and controlled water content, are strongly recommended to establish reliable structure–activity relationships. By addressing these challenges and focusing on these research directions, the field can advance toward a more mechanistically guided and rational development of catalytic systems for CO_2_ utilization via epoxide carbonation.

## Data Availability

The data supporting the findings of this study are available within the article, as it is a review article. Any additional data sets can be obtained from the corresponding authors upon reasonable request.

## ⊥

Universitdad de Murcia, Campus de Espinardo, Murcia E-30100, Spain
